# Molecular mechanisms of AMPK/YAP/NLRP3 signaling pathway affecting the occurrence and development of ankylosing spondylitis

**DOI:** 10.1186/s13018-023-04200-x

**Published:** 2023-11-04

**Authors:** Ruiyang Fu, Xiaoqing Guo, Zhongqiang Pan, Yaling Wang, Jing Xu, Lei Zhang, Jinxia Li

**Affiliations:** Department of Acupuncture and Tuina, Huzhou Hospital of Traditional Chinese Medicine, Affiliated to Zhejiang Chinese Medical University, Huzhou, 313000 Zhejiang Province People’s Republic of China

**Keywords:** Ankylosing spondylitis, AMPK, Phosphorylation activation, YAP, NLRP3, Ubiquitination degradation

## Abstract

**Background:**

Investigate the AMPK (protein kinase AMP-activated catalytic subunit alpha 1)/YAP (Yes1 associated transcriptional regulator)/NLRP3 (NLR family pyrin domain containing 3) signaling pathway's role in ankylosing spondylitis (AS) development using public database analysis, in vitro and in vivo experiments.

**Methods:**

Retrieve AS dataset, analyze differential gene expression in R, conduct functional enrichment analysis, collect 30 AS patient and 30 normal control samples, and construct a mouse model. ELISA, IP, and knockdown experiments were performed to detect expression changes.

**Results:**

NLRP3 was identified as a significant AS-related gene. Caspase-1, IL-1β, IL-17A, IL-18, IL-23, YAP, and NLRP3 were upregulated in AS patients. Overexpressing AMPK inhibited YAP's blockade on NLRP3 ubiquitination, reducing ossification in fibroblasts. Inhibiting AMPK exacerbated AS symptoms in AS mice.

**Conclusion:**

AMPK may suppress YAP expression, leading to NLRP3 inflammasome inhibition and AS alleviation.

**Supplementary Information:**

The online version contains supplementary material available at 10.1186/s13018-023-04200-x.

## Background

Ankylosing Spondylitis (AS) is a disease characterized by inflammation of the sacroiliac joints and attachment points of the spine [1]. In the early stage, ankylosing spondylitis (AS) mainly presents as inflammation and laboratory tests could detect elevated levels of C-reactive protein and erythrocyte sedimentation rate. As the disease progresses, it affects the spine, sacroiliac joints, hips, etc., resulting in spinal deformities, hip fusion, ossification of ligaments, forming a typical bamboo-like appearance, severely affecting the patient's daily life and mobility, with a high incidence of disability [2]. Epidemiological surveys show that the prevalence of ankylosing spondylitis (AS) in China is around 0.3%. However, given the huge population in China, there are quite a large number of AS patients. With a high prevalence and disability rate, AS is a serious public health problem [3, 4]. Patients with ankylosing spondylitis (AS) exhibit chronic inflammatory responses in their joints and entheses, leading to joint fusion and bone damage, ultimately affecting their quality of life and mobility. Additionally, the abnormal production and expression of inflammatory cytokines are considered crucial factors in the pathogenesis of AS [5]. The histopathological characteristics of AS are chronic inflammation of the synovium and ligament attachment sites, synovial cell proliferation, lymphoid infiltration, and the formation of vessel membranes, which leads to erosion and destruction of the bones and fibrosis and ossification of the connective tissues around the spine, eventually developing into spinal joint ossification and local osteoporosis [6, 7]. Fibroblasts are the main cells of connective tissue surrounding the joints. Under the stimulation of inflammatory mediators, fibroblasts secrete a large number of collagen fibers and bone matrix, resulting in pathological bone formation [8].

NLRP3 (NLR Family Pyrin Domain Containing 3) inflammasome, an important component of innate immunity, plays a vital role in the human body's immune response and disease development [9]. Due to its activation by multiple types of pathogens or danger signals, the NLRP3 inflammasome has played critical roles in various disease processes, being implicated in the onset and progression of diseases such as diabetes, obesity, rheumatoid arthritis, Alzheimer's disease, Parkinson's disease, and other autoimmune and inflammatory diseases [10]. NLRP3 inflammasome is a cytosolic protein complex that is a multimer and responds to cellular stress through assembling [11]. NLRP3 aggregates in the cytoplasm and interacts with adaptor protein ASC and cysteine protease family Caspase-1 to form a complex, promoting the self-activation of Caspase-1, the maturation of pro-inflammatory cytokines such as IL-1β, IL-18, as well as the death of inflammasomes and pyroptotic cells [11]. Although the regulatory mechanisms have gradually become clear after long-term research, the metabolic regulation of NLRP3 inflammasome remains a mystery. The occurrence of AS is closely related to the NLRP3 inflammasome axis-associated factors NLRP3, caspase-1, ASC, IL-1β, IL-17A, and IL-23 [12]. In the inflammatory response of arthritis, ATP plays a very important role. As the most important inflammatory factor, NLRP3 is activated and maintained by the mitochondrial electron transport chain through ATP production in NLRP3 inflammasome activation [13, 14].

YAP (Yes1 Associated Transcriptional Regulator) encodes the downstream nuclear effector of the Hippo signaling pathway, an important transcription factor that plays a key role in various biological processes related to cell proliferation, differentiation, and survival [15]. YAP may participate in the pathogenesis of AS through various mechanisms such as regulating inflammatory mediators, promoting osteoblast proliferation and differentiation, etc. In addition, some drugs and treatment strategies have been found to improve the symptoms and prognosis of autoimmune diseases by inhibiting the YAP signaling pathway [16, 17]. The expression level of YAP has significantly increased in inflammation and bone tissue reconstruction [18]. The Hippo signaling pathway, in which YAP participates, is also regulated by ATP changes. YAP could convert ATP to ADP. Moreover, studies have shown that AMPK regulates Hippo pathway activity to maintain energy homeostasis. The hippo pathway and AMP-activated protein kinase (AMPK) are activated during glucose starvation, leading to the phosphorylation and inactivation of YAP [19].

AMPK (AMP-activated protein kinase) is an important cellular energy sensor widely involved in regulating energy metabolism and biological processes such as protein synthesis in cells. In recent years, studies have shown that AMPK also plays an important role in inflammatory responses [20]. As a nutritional and energy sensor, it could sense the level of ATP in the cell. When mitochondria are suppressed under conditions of low-energy supply, AMPK phosphorylates specific enzymes and sites, which increases ATP production and reduces ATP consumption, thus restoring energy balance [21]. Studies have shown that AMPK could directly interact with YAP, thereby inhibiting YAP's transcriptional activity and function [22]. YAP could also participate in the inflammatory response by adjusting the expression of pro-inflammatory mediators such as IL-1β and IL-18 downstream of NLRP3 [23]. The YAP signaling pathway could enhance the ubiquitination modification of NLRP3, thereby reducing the degree of activation and inflammatory response of NLRP3 [24]. It is reported that AMPK could inhibit the activation of NLRP3 inflammasome, thereby reducing the production of IL-1β and IL-18 and thus suppressing inflammation [25].

In this study, we have identified that the AMPK/YAP/NLRP3 signaling pathway may play an important role in the occurrence and development of AS through bioinformatics analysis and experimental validation. Furthermore, we have revealed the relationship between inflammation and ossification, which provides new insights into treating inflammatory diseases. Our research will help deepen people's understanding of the pathological mechanism of AS, contribute to the early diagnosis and prediction of disease progression, and provide a theoretical basis for developing drugs targeting AMPK, which is expected to bring new treatment options for AS patients. In addition, the AS mouse model constructed in this study will provide an important tool for further investigating the pathogenesis of AS and evaluating the effectiveness of treatment strategies. Optimizing and applying this model could help improve the quality of research and the success rate of clinical translation.

## Materials and methods

### Transcriptome sequencing data

The RNA sequencing data of AS-related transcriptome were downloaded from the GEO (Gene Expression Omnibus) database (http://www.ncbi.nlm.nih.gov/geo/) using the chip GSE25101 with sequencing platform GPL6947 and the chip data from 5 healthy volunteers and 5 AS patients' peripheral blood samples were analyzed. Using the chip GSE13782 with sequencing platform GPL1261 we analyzed the chip data set from 3 normal mice and 3 AS mice intervertebral disc samples.

### Differential gene expression analysis with combined GO and KEGG pathway enrichment analysis

Using the GEO dataset, genes with |log FC|> 0.5 and *P *< 0.05 were selected as differentially expressed genes using the R package limma. All our analyses were conducted in R version 4.2.1 (R Foundation for Statistical Computing).

Using the clusterProfiler package in R software, Gene Ontology (GO) functional and Kyoto Encyclopedia of Genes and Genomes (KEGG) pathway enrichment analysis were performed on co-expressed genes, and visualization analysis of data was conducted through the ggplot2 package [26].

### Clinical sample

30 AS patients and 30 normal control peripheral blood samples were collected. All patients with AS are over 18 years old and diagnosed according to the EULAR criteria. There were 20 male and 10 female patients among those with AS; their ages ranged from 25 to 60 years old (with a mean age of 37.25 ± 10.17 years). Patients with severe lesions in the heart, brain, liver, kidney, or other important organs, as well as those with blood or endocrine system diseases, primary lesions outside the spine, psoriasis, inflammatory bowel disease, or uveitis, and those who were pregnant, breastfeeding, or diagnosed with acute eye inflammation requiring corticosteroid treatment, were excluded from the study. Among 30 healthy individuals, 15 males and 15 females ranged from 25 to 60 years (mean age 36.94 ± 10.65 years). They were included as healthy controls based on the following criteria: exclusion criteria for the aforementioned diseases, exclusion of diseases or symptoms of spinal joints, and exclusion of chronic or autoimmune diseases.

Collect peripheral blood samples from AS patients before treatment. Normal controls are healthy subjects matched for age and gender, and their health status is confirmed by medical examination. The clinical study was approved by the Clinical Review Committee of Huzhou Hospital of Traditional Chinese Medicine, Affiliated to Zhejiang Chinese Medical University (No. 2021-037-A), and all subjects have provided written informed consent forms. Use anticoagulated tubes to extract peripheral blood samples [27].

### Isolation and culture of mononuclear cells

Using heparin anticoagulant tubes, 25 mL of blood was extracted from patients with ankylosing spondylitis (AS) and healthy individuals, as well as from mice. The purpose of this blood collection was to isolate viable mononuclear cells [28].

Centrifuge the supernatant to collect mononuclear cells, add the mononuclear cell supernatant to the culture medium with a concentration of 5%, and co-culture with human synovial fibroblasts (HSF, tings-951131, Hefei Wanwu Biotechnology Co., Ltd.) for 48 h [29].

### Cell culture

HEK293T human kidney epithelial cell line (CBP60661, Nanjing Kai Bai biological technology Co., Ltd., Jiangsu, China), human synovial fibroblasts (HSF, tings-951131, Hefei Wanwu biological technology Co., Ltd.). The cells were cultured in RPMI 1640 medium (Gibco, Carlsbad, CA) containing 1% penicillin–streptomycin (10,378,016, Invitrogen) and 10% fetal bovine serum (FBS, Gibco, catalog number: 10100147, Invitrogen). They were placed in a cell culture incubator at 37 °C with 5% CO_2_. Change the growth medium every three days. When the culture reaches 80% confluency, it is passaged by digestion with 0.25% trypsin/EDTA (40127ES60, Yisheng Biotech, Shanghai, China) [30].

### Enzyme-linked immunosorbent assay (ELISA)

Serum acquisition: Peripheral blood from subjects and blood surrounding the eyes of mice were collected. After coagulation at room temperature for 20–30 min, the blood was centrifuged at 2000 × g for 10 min to obtain serum samples. Then store the serum samples at − 80 °C. The levels of caspase-1, interleukin-1β (IL-1β), interleukin-17A (IL-17A), interleukin-18 (IL-18), and interleukin-23 (IL-23) were measured using the following ELISA kits: caspase-1 ELISA kit (EH70RB, human, Thermo Fisher), caspase-1 ELISA kit (kt21346, mouse, MSK), IL-1β ELISA kit (kt98060, human, MSK), IL-1β ELISA kit (kt21178, mouse, MSK), IL-17A ELISA kit (kt99222, human, MSK), IL-17A ELISA kit (kt21287, mouse, MSK), IL-18 ELISA kit (kt98065, human, MSK), IL-18 ELISA kit (kt21183, mouse, MSK), IL-23 ELISA kit (kt98055, human, MSK), and IL-23 ELISA kit (kt21102, mouse, MSK) [31].

### RT-qPCR

Cells were lysed using Trizol reagent (catalog number: 10296010, Invitrogen, ThermoFisher, USA) to extract total cellular RNA. RNA quality and concentration were determined using UV–visible spectrophotometry (ND-1000, Nanodrop, USA).

To detect mRNA expression, PrimeScript™ RT-qPCR Kit (Cat. No.: RR086A, TaKaRa, Mountain View, CA, USA) was used for reverse transcription. In addition, real-time quantitative reverse transcription polymerase chain reaction (RT-qPCR) was performed on a LightCycler 480 system (Roche Diagnostics, Pleasanton, CA, USA) using SYBR Premix Ex TaqTM (Cat. No. DRR820A, TaKaRa). Using GAPDH as the internal reference for mRNA analysis. The primers used for amplification were designed and provided by Shanghai General Biosciences Co., Ltd. The primer sequences are shown in Additional file 1: Tables S1 and S2. 2-ΔΔCt represents the relative expression ratio of the target gene in the experimental group compared to the control group, and the formula is as follows: ΔΔCT = ΔCt experimental group—ΔCt control group, where ΔCt = target gene Ct—reference gene Ct [32].

### Western blot

Extract total protein from human peripheral blood and mouse spinal tissue. Digest and collect cultured cells using trypsin, lyse the cells using an enhanced RIPA lysis buffer containing a protease inhibitor (AR0108, Dr. De in Wuhan, Hubei, China) and measure the protein concentration using a BCA protein quantification kit (catalog number: AR1189, Dr. De in Wuhan, Hubei, China). Proteins were separated using SDS-PAGE, transferred to PVDF membrane after separation, blocked with 5% BSA at room temperature for 1 h, and then incubated separately with diluted primary antibodies including p-AMPK (Cat #50,081, 1/1000; CST, human and mouse, CellSignal), AMPK (#5831, 1/1000; CST, human and mouse), p-YAP (#13,008, 1/1000; CST, human and mouse), YAP (#12,395, 1/1000; CST, human and mouse), NLRP3 (#15,101, 1/1000; CST, human and mouse), GAPDH (G9545, 1/2000; Sigma, human and mouse, Sigma-Aldrich), Myc-tag (#2272, 1/1000; CST, human and mouse), HA-tag (#2367, 1/1000; CST, human and mouse), Flag-tag (MA1-91,878, 1/1000; Sigma, USA) at 4 °C overnight. Wash the membrane three times with PBST (3 × 5 min). Then, add Anti-Mouse-HRP secondary antibody (Cat # 7076, 1/5000; CST) or Anti-Rabbit-HRP secondary antibody (Cat # 7074, 1/5000; CST) and incubate at room temperature for 1 h. Finally, wash the membrane three times with PBST (3 × 5 min). Discard PBST and add the appropriate ECL working solution (Omt-01, Beijing Aomijia De Medical Technology Co., Ltd., Beijing, China). Incubate the transfer membrane at room temperature for 1 min, then remove excess ECL reagent, seal with plastic wrap, and expose to X-ray film in a dark box for 5–10 min for developing and fixing. Quantify the gray values of each band in Western blot images using Image J software, with GAPDH as an internal reference [33, 34].

### Measurement of ATP, AMP, and detection of mitochondrial ROS

According to the respective manufacturer's standard instructions, use an ATP assay kit (High-Performance Liquid Chromatography) (BC0304, Beijing Solabao, China) or an AMP content detection kit (High-Performance Liquid Chromatography) (BC1024, Beijing Solabao) to measure ATP or AMP levels in human and mouse peripheral blood mononuclear cells using High-Performance Liquid Chromatography equipment (SCIEX Triple Quad™ 3500). Then, calculate the content through a standard curve [35].

The MitoSOX red mitochondrial superoxide indicator (M36008, Thermo Fisher, USA) is used to identify mitochondrial reactive oxygen species (mROS) in human and mouse peripheral blood mononuclear cells. Cells are incubated with MitoSOX (25 μM) in PBS at 37 °C for 30 min. Observations were made using a laser confocal microscope (magnification, × 600; STELLARIS 5 Cryo, Leica) [36].

### Mitochondrial respiratory chain complex detection

According to the standard instructions of the respective manufacturers, the activity of mitochondrial respiratory chain complexes I (NADH-coenzyme Q reductase), II, III (ubiquinol-cytochrome c reductase) and IV (cytochrome c oxidase) could be sequentially detected in human or murine mononuclear cells by using the mitochondrial respiratory chain complex I (NADH-coenzyme Q reductase) colorimetric assay kit (E-BC-K149-M), mitochondrial respiratory chain complex II colorimetric assay kit (E-BC-K150-M), mitochondrial respiratory chain complex III colorimetric assay kit (E-BC-K151-M), and mitochondrial respiratory chain complex IV colorimetric assay kit (E-BC-K152-M) from Wuhan Elabscience Biotechnology Co., Ltd. in Hubei, China. The detection involves the addition of buffer, reaction, and substrate solutions, followed by measurement of the activity of mitochondrial respiratory chain complexes I-IV at different wavelengths (mitochondrial respiratory chain complex I: 340 nm; mitochondrial respiratory chain complex II: 600 nm; mitochondrial respiratory chain complex III: 550 nm; mitochondrial respiratory chain complex IV: 550 nm) using a microplate reader.

### Ubiquitin-mediated immunoprecipitation

Transfect Myc-NLRP3, HA-Ub, GFP-YAP, Flag-AMPK plasmids (all purchased from Shanghai Bioengineering) and proteasome inhibitor MG132 (0.5, 1.0, 2.0 μmol/L) (Y-13259, MCE, Shanghai, China) of different concentrations into HEK293T cells, add Protein A/G agarose beads (ab193262, Abcam) to remove non-specific proteins, then immunoprecipitate using anti-Myc antibody (#2272, 1/1000; CST, human and mouse). Next, wash the co-immunoprecipitated samples multiple times to remove non-specific proteins and impurities, retaining the ubiquitinated protein and its interacting protein complex. Then, use anti-Myc antibody (#2272, 1/1000; CST, human and mouse), anti-Flag antibody (MA1-91878, 1/1000; Sigma, USA), anti-HA antibody (#2367, 1/1000; CST, human and mouse), and anti-GFP antibody (#2956, 1/1000; CST, human and mouse) for immunoblotting detection [24].

### Cell grouping and infection

The human NLRP3-shRNA (sequence 1, 5′-3′: CCGTAAGAAGTACAGAAAGTA; sequence 2, 5′-3′: CCAGCCAGAGTCTAACTGAAT) was purchased from Sigma-Aldrich (Shanghai, China); the human YAP-shRNA (sequence 1, 5′-3′: CCCAGTTAAATGTTCACCAAT; sequence 2, 5′-3′: GCCACCAAGCTAGATAAAGAA) was purchased from Sigma-Aldrich (Shanghai, China); the sh-NC (sequence, 5′-3′: GCAACAAGATGAAGAGCACCAA) was purchased from Sigma-Aldrich (Shanghai, China). Mouse NLRP3-shRNA (sequence 1, 5′-3′: CCATACCTTCAGTCTTGTCTT; sequence 2, 5′-3′: CCGGCCTTACTTCAATCTGTT) was purchased from Sigma-Aldrich (Shanghai, China); Mouse YAP-shRNA (sequence 1, 5′-3′: CGGTTGAAACAACAGGAATTA; sequence 2, 5′-3′: GCGGTTGAAACAACAGGAATT) was purchased from Sigma-Aldrich (Shanghai, China); sh-NC (sequence, 5′-3′: CCTAAGGTTAAGTCGCCCTCG) was purchased from Sigma-Aldrich (Shanghai, China).

Cell lines targeting NLRP3 (sh-NLRP3-LTEP-s, referred to as sh-NLRP3) and YAP (sh-YAP-LTEP-s, referred to as sh-YAP), as well as a control cell line (sh-NC-LTEP-s, referred to as sh-NC), were established based on HEK293 cells through slow virus transduction. Plasmids and slow virus packaging services were provided by Sengong Biotech (Shanghai, China). The constructed plasmids containing single luciferase reporter gene(sh-NC-luc, sh-NLRP3-luc, sh-YAP-luc, and oe-NC-luc) were co-transfected with helper plasmids into 293 T cells. After verification, amplification, and purification, packaged lentivirus was obtained. 5 × 10^5^ cells were seeded into 6-well plates for cell transfection mediated by lentivirus. When the confluence reached 70–90%, the medium containing an appropriate amount of packaged lentivirus (MOI = 10, working titer of approximately 5 × 10^6^ TU/mL) and 5 μg/mL polybrene (Merck, TR-1003, USA) was added for transfection. After 4 h, an equal amount of fresh medium was added to dilute the polybrene. After 24 h of transfection, the medium was replaced with a fresh one. After 48 h, the reporter gene assay observed the transfection efficiency and stable cell lines were selected using appropriate concentrations of puromycin (A1113803, Gibco, Grand Island, NY, USA). Collect cells when they no longer die in the purine mine culture medium and confirm the knockdown efficiency through RT-qPCR [37].

Drug treatment doses for cells: Cells were treated with 10 μM BAY-3827 (S9833, Selleck, Shanghai, China) for 48 h and 5 mM Metformin (HY-B0627, MCE, Shanghai, China) for 48 h.

Mononuclear cell groups: sh-NC + AS group (mononuclear cells from AS patients infected with sh-NC lentivirus), sh-YAP + AS group (mononuclear cells from AS patients infected with sh-YAP lentivirus), sh-NLRP3 + AS group (mononuclear cells from AS patients infected with sh-NLRP3 lentivirus), BAY-3827 + AS group (mononuclear cells from AS patients treated with BAY-3827), metformin + AS group (mononuclear cells from AS patients treated with metformin), BAY-3827 + sh-YAP + AS group (mononuclear cells from AS patients infected with sh-YAP lentivirus and treated with BAY-3827), BAY-3827 + sh-NLRP3 + AS group (mononuclear cells from AS patients infected with sh-NLRP3 lentivirus and treated with BAY-3827).

Fibroblast Grouping: sh-NC + AS-M-CM (AS patient monocyte cell media) group (co-cultured with fibroblasts and the supernatant of monocytes derived from AS patients infected with sh-NC lentivirus), sh-YAP + AS-M-CM group (co-cultured with fibroblasts and the supernatant of monocytes derived from AS patients infected with sh-YAP lentivirus), sh-NLRP3 + AS-M-CM group (co-cultured with fibroblasts and the supernatant of monocytes derived from AS patients infected with sh-NLRP3 lentivirus), BAY-3827 + AS-M-CM group (co-cultured with fibroblasts and the supernatant of AS patient monocytes treated with BAY-3827), metformin + AS-M-CM group (co-cultured with fibroblasts and the supernatant of AS patient monocytes treated with metformin), BAY-3827 + sh-YAP + AS-M-CM group (co-cultured with fibroblasts and the supernatant of AS patient monocytes treated with BAY-3827 and infected with sh-YAP lentivirus), BAY-3827 + sh-NLRP3 + AS-M-CM group (co-cultured with fibroblasts and the supernatant of AS patient monocytes treated with BAY-3827 and infected with sh-NLRP3 lentivirus).

### Apoptosis-associated speck-like protein containing CARD (ASC) speckles.

Macrophages were stimulated with LPS (100 ng/mL) (SMB00610, Sigma-Aldrich) for 3 h, followed by stimulation with 10 μM nigericin (Nig) (S25116, Genewiz, Shanghai, China) for 1 h to activate NLRP3 inflammasome. Cells were then fixed in 4% paraformaldehyde and permeabilized with 0.1% Triton X-100 for 30 min. Then seal the slides with phosphate-buffered saline (PBS) containing 1% bovine serum albumin (BSA), followed by staining with ASC and DAPI. Observe cells through a fluorescence microscope [24].

### Alkaline phosphatase (ALP) activity assay

By using ALP quantitative assay kit (P0321S, BiyunTian, Shanghai, China), ALP activity could be determined by measuring the phenol produced from the catalytic decomposition of sodium phenyl phosphate as the substrate of ALP, combined with protein concentration and calculated by a formula. First, configure the ALP assay kit according to the instructions: dilute 1 mg/mL phenol standard solution to 0.1 mg/mL standard working fluid. Next, add 5 μL of double-distilled water to the blank tube, 5 μL of phenol standard application fluid to the standard tube, and 5 μL of protein supernatant to the test tube. Next, add 50 μL of buffer solution and 50 μL of substrate solution in each EP tube in sequence, mix well, and incubate at 37 °C water bath for 15 min. Finally, transfer the liquid to a 96-well plate and add 150 μL of the color developer to each well. Be careful not to produce bubbles to avoid affecting the enzyme-labeled instrument measurement. After mixing, measure the absorbance OD value at 520 nm wavelength in the enzyme-labeled instrument and obtain data for analysis [38].

### ALP and Eosin staining

Use an alkaline phosphatase colorimetric kit (CTCC-JD002, Puhe BioTech, Wuxi, China) to detect ALP. According to the instructions, prepare ALP staining solution. Add 33 μL of BCIP solution and 66 μL of NBT solution to each 10 mL color development buffer. After mixing evenly, add an appropriate amount of staining solution to each well to cover the cells. Incubate at room temperature in the dark for 30 min. If no blue staining is observed after 30 min, continue incubating overnight and observe again. Discard the supernatant after dyeing and wash twice with distilled water to terminate the reaction. Take photos after discarding the supernatant [39].

Detection was carried out using a Safranin O staining kit (C01383, Shanghai Yajie Biotechnology, Shanghai, China). The principle is that after the chelation of Alizarin red with calcium ions, a visible orange-red complex is formed, which is used to determine whether there are calcium nodules. The specific operation steps are: add an appropriate amount of Alizarin red staining solution, cover the cells, and incubate at room temperature for 5 min. The orange-red lumps could be seen in the pores. Then, discard the clear staining solution, rinse with double-distilled water three times to terminate the reaction, and take a photo [38].

### Construction of AS mouse model

The experimental animals used in this study were 24–26 week-old male BALB/c mice purchased from Cyagen Biosciences Inc (Suzhou, China). The mice were raised in an SPF-level animal room. All animals have been approved by the Ethics Committee of Huzhou Hospital of Traditional Chinese Medicine, Affiliated to Zhejiang Chinese Medical University. But, first, anesthetize using intraperitoneal injection of 1% pentobarbital sodium solution (40 mg/kg).

Using proteoglycan to induce BALB/c mice, take 100 μL of immunoadjuvant (Dimethyldioctadecylammonium bromide, DDA) suspension (mix 3300 μL of sterile PBS with 33 mg of DDA) and 100 μL of proteoglycan solution (P5864-10MG, Sigma-Aldrich) (dissolved in PBS, the concentration of 10 mg/mL), invert several times to mix well, and the resulting 200 μL mixture could be used for injection. Inject BALB/c mice aged 24–26 weeks with 200 μL injection dose for the spine. The first injection is remembered as Week 0, and the subsequent two boost injections are given at Week 3 and Week 6, respectively. Afterward, observe the mice three times a week and record any abnormal changes caused by arthritis change.

Two weeks after the third injection of chondroitin sulfate, redness, and swelling could be observed on the paws of mice. Usually, spinal involvement occurs after the third injection and 8 weeks later, at which time the spine's intervertebral disc will be eroded. Therefore, after 18 weeks, we euthanized the mice using the dislocation of the cervical vertebrae method, dissected and cleaned the spinal column, performed a CT scan to assess the changes in bone structure, and determined the success of the induced model [40].

Seven days after successful AS modeling, the slow virus was injected into the tail vein, with a working titer of 5 × 106 TU/mL and an injection dose of 10 μg per mouse for continuous injection for one week. In addition, seven days after the successful AS modeling, BAY-387 was injected via the tail vein at a dose of 0.02 mg/kg for one week. Also, seven days after the successful AS modeling, metformin was injected via the tail vein at a 5 mg/kg dose for one week.

Mouse grouping: divided into normal mouse group and AS model mouse group according to whether AS is generated, with 6 mice in each group; based on AS modeling, divided into DMSO + AS group, sh-NC + AS group, BAY-387 + AS group, metformin + AS group, sh-YAP + AS group, sh-NLRP3 + AS group, BAY-387 + sh-YAP + AS group, and BAY-387 + sh-NLRP3 + AS group, with 6 mice in each group.

### In vivo* micro-computed tomography (micro-CT)*

AS mice that were successfully induced were euthanized by cervical dislocation, and their spines were dissected and immediately placed in 10% neutral buffered formalin and then incubated for 2 weeks. The samples were imaged using a desktop cone-beam micro-computed tomography (micro-CT) scanner (LASER6000, Guangzhou Light Instrument Co., Ltd., Guangzhou, China). The imaging parameters were set as follows: resolution of 12 million pixels, pixel density of 16 bits (0–65536 colors), filtered back projection algorithm (NRecon software; Bruker microCT, Kontich, Belgium), pixel size of 4.63 μm × 4.63 μm, dynamic range greater than 4.8 orders of magnitude, and an electric lens with F = 0.95 and a 35 mm electric lens. The images were reconstructed using software to produce cross-sectional images with a pixel size of 16 μm.[41].

### Histological staining

After the AS model was established, the mice were killed using cervical dislocation, and the spinal cord was removed with tissue scissors. Bloodstains were washed off with PBS, and the specimens were fixed in 4% paraformaldehyde for 48 h. Then, they were decalcified in 10% EDTA for 4 weeks, embedded, and sliced.

Su-Mu-Jing and H&E staining: Take paraffin sections of tissue, dewax with xylene, dehydrate gradually with ethanol, and stain with su-mu-jing (H8070, Beijing Solabo Technology Co., Ltd., Beijing, China) for 5 min, immerse in hematoxylin and eosin (G1100, Beijing Solabo Technology Co., Ltd., Beijing, China) solution for 2–3 min, dehydrate conventionally, transparentize, and seal the slides.

Hematoxylin and eosin staining (G1371, Beijing Soleibao Technology Co., Ltd., Beijing, China): Paraffin sections were dewaxed in water, counterstained with hematoxylin, stained with 0.02% eosin for 3–5 min, rinsed in water to remove excess dye until the cartilage appeared colorless, and then stained with 0.1% safranin (stained for 1–2 min, rinsed in water to remove excess dye), dehydrated in 95% and absolute ethanol, cleared in xylene, and finally mounted with neutral resin [42].

### Immunohistochemistry

The organization or cells to be detected need to be obtained, fixed, and embedded. Cut the embedded organization into thin slices and perform the dewaxing treatment. De-waxing removes wax from the tissue slice, making it hydrophilic and facilitating subsequent immunostaining operations. Treated with the specific Vimentin antibody (#46,173, 1/1000; CST, human and mouse) and Anti-Rabbit-HRP secondary antibody (#8114, 1/5000; CST) for the deparaffinized tissue sections. Use DAB staining reagent (ab64238, Abcam, Shanghai, China) to visualize the binding site between the secondary and primary antibodies. The tissue slices that have been stained are processed to remove the wax and then sealed for observation. Observe slices under a microscope and record the expression of Vimentin [43].

### Statistical analysis

All experiments were independently conducted at least three times, and the data were presented as mean ± standard deviation. Use paired-sample t-test or independent-sample t-test to analyze the differences between groups. All statistical analysis was performed using GraphPad Prism 5.0. When the *P* value is less than 0.05, it is considered statistically significant [44].

## Results

### Bioinformatics analysis confirmed that the AMPK/YAP/NLRP3 signaling pathway may play an important role in the occurrence and development of AS

**Fig. 1:**
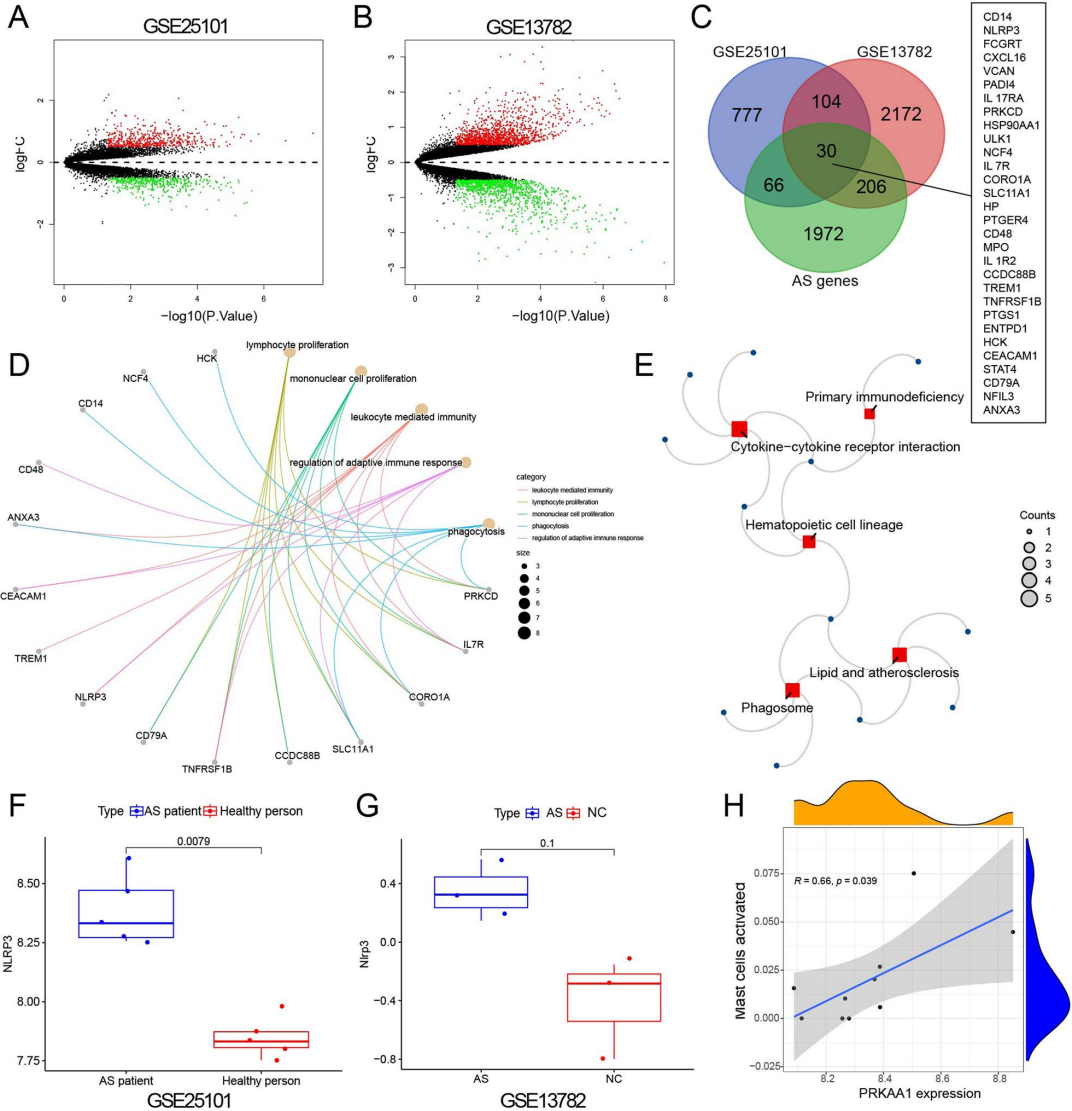
Bioinformatics analysis suggests the involvement of the AMPK/YAP/NLRP3 signaling pathway in the occurrence and development of ankylosing spondylitis (AS). *Note* **A** Volcano plot of differentially expressed genes in GSE25101 dataset (AS patient group: n = 5; Healthy person group: n = 5), with red dots representing significantly upregulated genes, green dots representing significantly downregulated genes, and black dots representing genes with no significant difference. **B** Volcano plot of differentially expressed genes in the GSE13782 dataset (AS group: n = 3; NC group: n = 3), with red dots representing significantly upregulated genes, green dots representing significantly downregulated genes, and black dots representing genes with no significant difference. **C** Venn diagram of the intersection of differentially expressed genes in GSE25101, GSE13782 datasets, and AS-related genes. **D**—KEGG **E** Functional enrichment analysis of 30 differentially expressed genes. **F** Box plot of NLRP3 differential expression in GSE25101 dataset (AS patient group: n = 5; Healthy person group: n = 5). **G** Box plot of NLRP3 differential expression in GSE13782 dataset (AS group: n = 3; NC group: n = 3). **H** Correlation analysis of AMPK (PRKAA1) with Mast cells

To identify key genes that function in AS, we downloaded the GSE25101 and GSE13782 microarray chips of AS-related transcriptome RNA sequencing data from the GEO database. The GSE25101 chip analyzed RNA from 5 active AS patients and RNA from 5 gender and age-matched control groups, identifying 997 differentially expressed genes, including 578 upregulated genes and 419 downregulated genes (Fig. 1A). GSE13782 chip analyzed the RNA of 3 normal mice and 3 AS mice induced by human chondroitin sulfate protein and screened out 2512 differentially expressed genes, including 1369 up-regulated genes and 1143 down-regulated genes (Fig. 1B). The intersection of differential genes between two chip data and 2274 AS-related genes (obtained from the GeneCards database) yielded 30 intersecting genes (Fig. 1C). Then, the 30 differentially expressed genes were subjected to GO and KEGG enrichment analysis using both chips. GO analysis revealed that they were mainly associated with lymphocyte proliferation, mononuclear cell proliferation, leukocyte-mediated immunity, and regulation of adaptive immune responses (Fig. 1D). KEGG analysis revealed their association with primary immunodeficiency pathways (Fig. 1E).

AS is an autoimmune inflammatory disease, so this study focuses on the inflammatory immune response. Among the 30 differential genes, NLRP3 is an important inflammatory factor that is central to innate immunity [45]. Therefore, this study aims to explore the pathway that affects AS by focusing on NLRP3 (Fig. 1F, G). ATP is released and involved in the activation of inflammatory cells and the release of inflammatory mediators. ATP has been reported as an activation factor of NLRP3. Therefore, a decrease in ATP levels could activate NLRP3 [13, 14, 46].

An increase in the AMP/ATP ratio activates AMPK, which inhibits ATP consumption pathways and increases ATP generation [21]. Previous studies have found that YAP could block the ubiquitination of NLRP3 in mouse macrophages, thereby increasing its expression level [24]. Furthermore, YAP is closely associated with immune and inflammatory diseases [47, 48]. TLR could activate the Hippo pathway in immune response [48]. Studies have shown (Fig. 1H) that simultaneous activation of the AMP-activated protein kinase (AMPK) pathway could suppress the expression of YAP [49]. Therefore, we speculate that the AMPK/YAP/NLRP3 signaling pathway may play an important role in the occurrence and development of AS.

### Levels of pro-inflammatory cytokines, p-AMPK, and NLRP3 in the peripheral blood samples of patients with AS were significantly increased

**Fig. 2:**
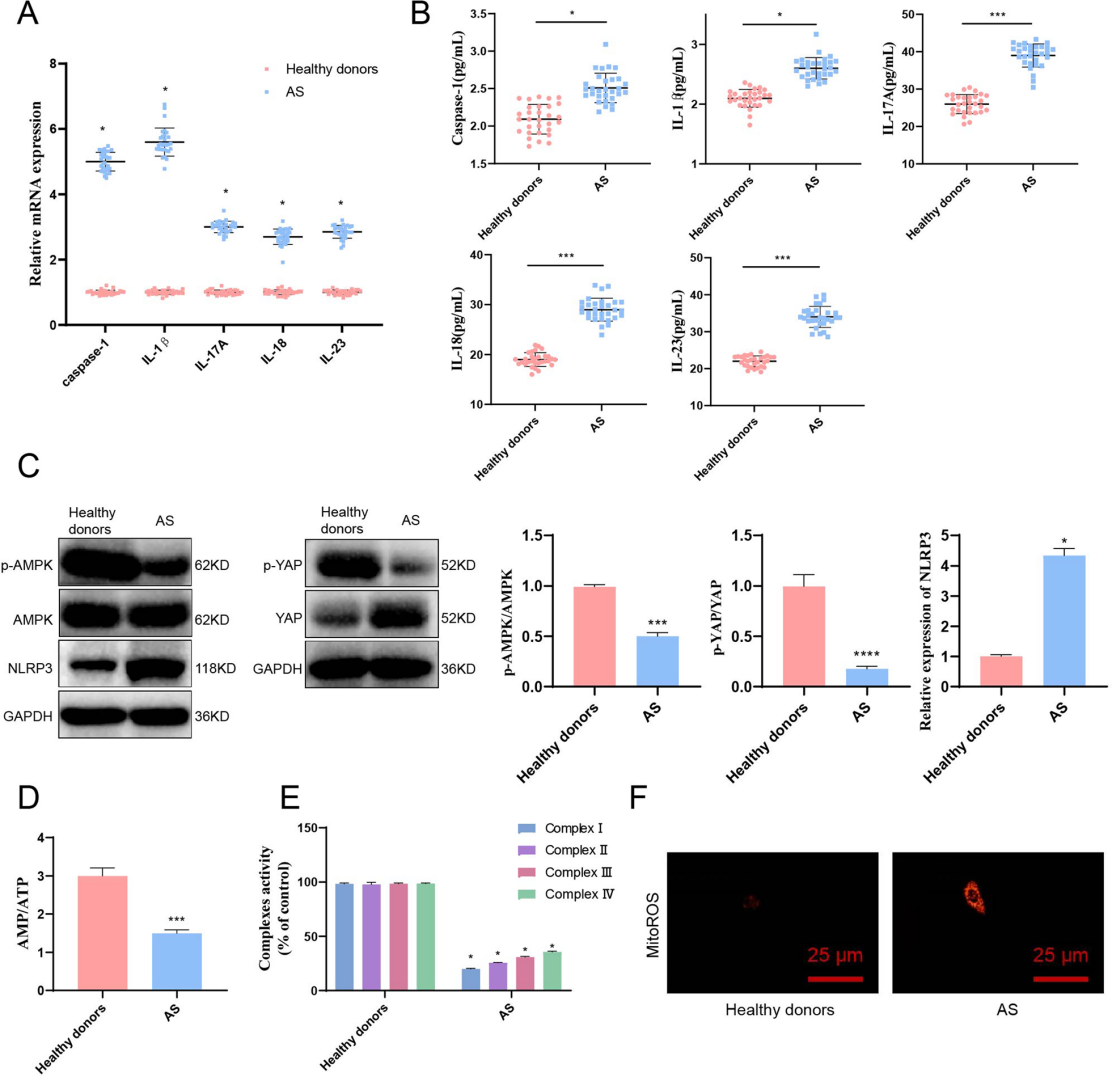
Differential expression of AMPK, YAP, and NLRP3 in peripheral blood of AS patients and healthy controls. *Note* **A** RT-qPCR was performed to detect the mRNA expression levels of caspase-1, IL-1β, IL-17A, IL-18, and IL-23 in peripheral blood of AS patients and healthy individuals; **B** ELISA was conducted to measure the levels of inflammatory factors caspase-1, IL-1β, IL-17A, IL-18, and IL-23 in the peripheral blood serum of AS patients and healthy individuals; **C** Western blot analysis was employed to assess the protein expression levels of AMPK, YAP, and NLRP3 in PBMCs from AS patients and healthy individuals; **D** The AMP/ATP ratio was determined in PBMCs from AS patients and healthy individuals; **E** The activities of mitochondrial complexes I-IV in PBMCs from AS patients and healthy individuals were measured; **F** Mitochondrial ROS in PBMCs from AS patients and healthy individuals were observed under a laser confocal microscope, with Bar = 10 μm. Numerical values are presented as mean ± standard deviation. All cell experiments were performed in triplicate. *indicates *P *< 0.05 and ***, ****indicates *P *< 0.001 and *P *< 0.0001, respectively, for comparing the two groups (Healthy donors = 30, AS = 30)

To determine the relevance of AS with NLRP3, YAP, and AMPK, we collected peripheral blood from AS patients and healthy controls, separated serum, extracted RNA, and detected the mRNA expression levels of caspase-1, IL-1β, IL-17A, IL-18, and IL-23 using ELISA and RT-qPCR (Fig. 2A, B). The results showed that caspase-1, IL-1β, IL-17A, IL-18, and IL-23 were significantly increased.

Using the collected peripheral blood mononuclear cells (PBMCs) obtained above, protein expression levels of AMPK, YAP, and NLRP3 were detected by Western blot (Fig. 2C). The results indicated that compared with the control group, p-AMPK/AMPK and p-YAP/YAP were significantly decreased in AS patients, indicating that the phosphorylation of AMPK and YAP were both inhibited.

AMPK/YAP/NLRP3 are all related to ATP. Therefore, the decrease in ATP level could activate NLRP3, while the increase in AMP/ATP ratio could activate AMPK. We simultaneously detected the contents of AMP and ATP in PBMCs and found that the AMP/ATP ratio of AS patients was significantly decreased compared with healthy individuals (Fig. 2D). In addition, AS inhibited the activity of mitochondrial complexes (Fig. 2E). Mitochondria generate superoxide during ATP production. When mitochondrial function is abnormal, excessive reactive oxygen species (ROS) accumulation could cause various cell functional disorders. We detected mitochondrial ROS in AS monocytes and found that compared to normal individuals, mitochondrial ROS in AS monocytes significantly increased (Fig. 2F).

Thus, we could preliminarily observe that pro-inflammatory factors are significantly increased in the peripheral blood of AS patients, and the AMPK signaling pathway is inhibited, inhibiting the phosphorylation of YAP. In contrast, the expression of NLRP3 is significantly increased.

### AMPK inhibits YAP and promotes NLRP3 K27 ubiquitination and degradation to suppress inflammasome activation

**Fig. 3:**
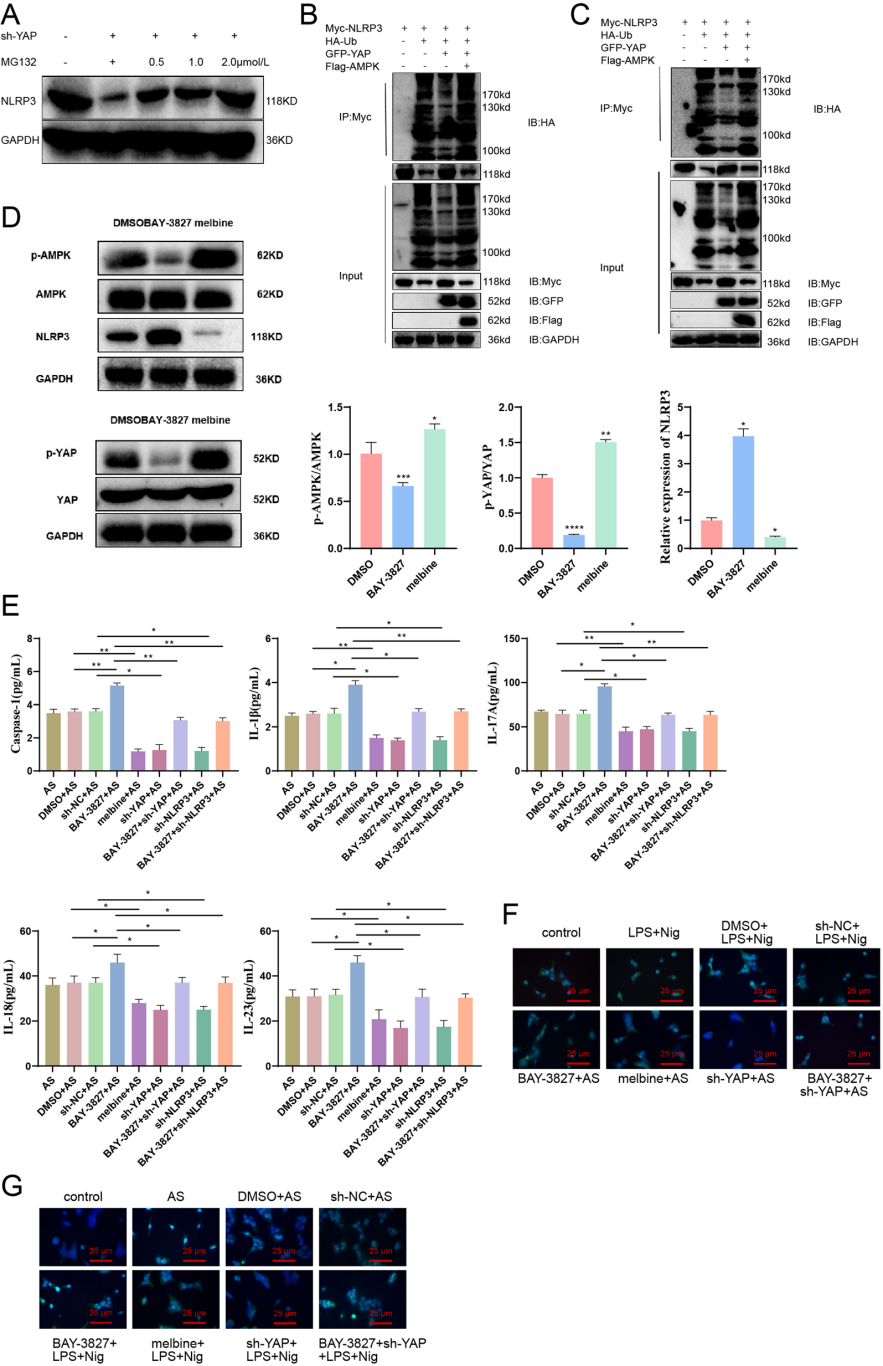
The impact of AMPK on NLRP3 K27 ubiquitination and inflammasome activation regulated by YAP. *Note* **A** Ubiquitinated immunoprecipitation was performed to detect the ubiquitination bands of NLRP3; **B** Ubiquitinated immunoprecipitation was conducted to examine the ubiquitination bands of NLRP3; **C** Ubiquitinated immunoprecipitation was employed to assess the ubiquitination bands of NLRP3; **D** Western blot analysis was used to measure the expression levels of AMPK, YAP, and NLRP3 in monocytes; **E** ELISA was performed to measure the levels of caspase-1, IL-1β, IL-17A, IL-18, and IL-23 in peripheral blood monocytes; **F**, **G** Formation of ASC specks in monocytes from healthy individuals (**F**) and AS patients **G** was observed through fluorescence microscopy. ASC: green, nucleus: blue, white arrows indicate ASC specks, Bar = 10 μm. Values are presented as mean ± standard deviation. All cell experiments were repeated three times. *Indicates *P *< 0.05 for comparison between two groups, **Indicates *P *< 0.01, and ***Indicates *P *< 0.001

In the above experiment, we found that the AMPK signaling pathway was inhibited in AS, the phosphorylation of YAP was also inhibited, and the expression of NLRP3 was increased. Previous studies have shown that AMPK could directly mediate the phosphorylation of YAP, thereby inhibiting the activation of the YAP pathway [22]. YAP could promote the activation of NLRP3 inflammasome by blocking the K27 ubiquitination of NLRP3 [24]. We co-transfected sh-YAP, Myc-NLRP3, and HA-ubiquitin (HA-Ub) plasmids to further investigate the relationship among the three into HEK293T cells. We treated them with proteinase inhibitor MG132 at different concentrations (0.5, 1.0, 2.0 μmol/L). We then used an HA antibody to detect the ubiquitinated bands of NLRP3. Our experimental results show that in the absence of MG132, sh-YAP could promote the ubiquitination of NLRP3. However, with the increase of MG132 concentration, the level of ubiquitination of NLRP3 gradually decreases. This result indicates that the degradation of NLRP3 is related to the proteasome (Fig. 3A).

Transfect Myc-NLRP3, HA-Ub, GFP-YAP, and Flag-AMPK plasmids into HEK293T cells, followed by immunoprecipitation with anti-Myc antibody and immunoblotting with anti-HA antibody to detect ubiquitinated NLRP3. Our experimental results showed that YAP could inhibit the ubiquitination of NLRP3, and overexpression of AMPK could block the inhibition of YAP on the ubiquitination of NLRP3 (Fig. 3B). Meanwhile, we replaced HA-Ub with HA-Ub-K27, labeled with HA and linked with K27 of ubiquitin, for ubiquitination detection in the above experiments. The results also indicate that YAP could suppress the ubiquitination of NLRP3 and overexpression of AMPK could block YAP's inhibition of the ubiquitination of NLRP3 (Fig. 3C). In addition, these results indicate that AMPK could inhibit YAP-mediated K27 ubiquitination of NLRP3.

We constructed two human shRNA sequences (sh-YAP-1 and sh-YAP-2) for YAP knockdown. Using RT-qPCR, we detected the knockdown efficiency in monocytes and found that sh-YAP-1 exhibited higher knockdown efficiency. Therefore, sh-YAP-1 was selected for subsequent experiments (hereafter referred to as sh-YAP) (Additional file 2: Fig. S1A). In addition, two human sh-RNA sequences targeting NLRP3 (sh-NLRP3-1 and sh-NLRP3-2) were detected for knockdown efficiency in monocytes using RT-qPCR technology. The results showed that the knockdown efficiency of sh-NLRP3-2 was higher. Therefore, sh-NLRP3-2 (hereafter referred to as sh-NLRP3) was selected for further experiments (Additional file 2: Fig. S1B).

We treated single-cell organisms with AMPK inhibitors (BAY-3827) and AMPK activators (metformin) and used Western blot to detect the expression levels of AMPK, YAP, and NLRP3. The results showed that BAY-3827 inhibited AMPK activation and YAP phosphorylation while increasing NLRP3 expression. Conversely, Metformin significantly activated AMPK activation and YAP phosphorylation while significantly reducing NLRP3 expression (Fig. 3D).

We divided the peripheral blood mononuclear cells of AS patients into the following groups: DMSO (control) group, AMPK inhibitor (BAY-3827) group, AMPK activator (metformin) group, sh-NC group, sh-YAP group, BAY-3827 + sh-YAP group, sh-NLRP3 group, BAY-3827 + sh-NLRP3 group. After centrifugation of the collected cell supernatant 48 h later, caspase-1, IL-1β, IL-17A, IL-18, and IL-23 were examined. The results showed that in the BAY-3827 group, the levels of pro-inflammatory factors increased significantly. In the metformin group, the levels of pro-inflammatory factors decreased significantly. In the sh-YAP and sh-NLRP3 groups, the levels of pro-inflammatory factors decreased significantly. Compared with the BAY-3827 group, the levels of pro-inflammatory factors decreased significantly in the BAY-3827 + sh-YAP and BAY-3827 + sh-NLRP3 groups (Fig. 3E–G).

ASC specks could serve as biomarkers for NLRP3 inflammasome. We could induce the activation of NLRP3 inflammasome by treating normal control mononuclear cells with LPS and Nig [24]. Knocking down YAP could inhibit ASC speck formation, while sh-YAP could suppress inflammasome activation induced by BAY-3827. AMPK inhibits the activation of NLRP3 inflammasome.

### Inhibition of AMPK could stimulate monocytes to secrete inflammatory factors and thus promote osteogenesis of fibroblasts

**Fig. 4:**
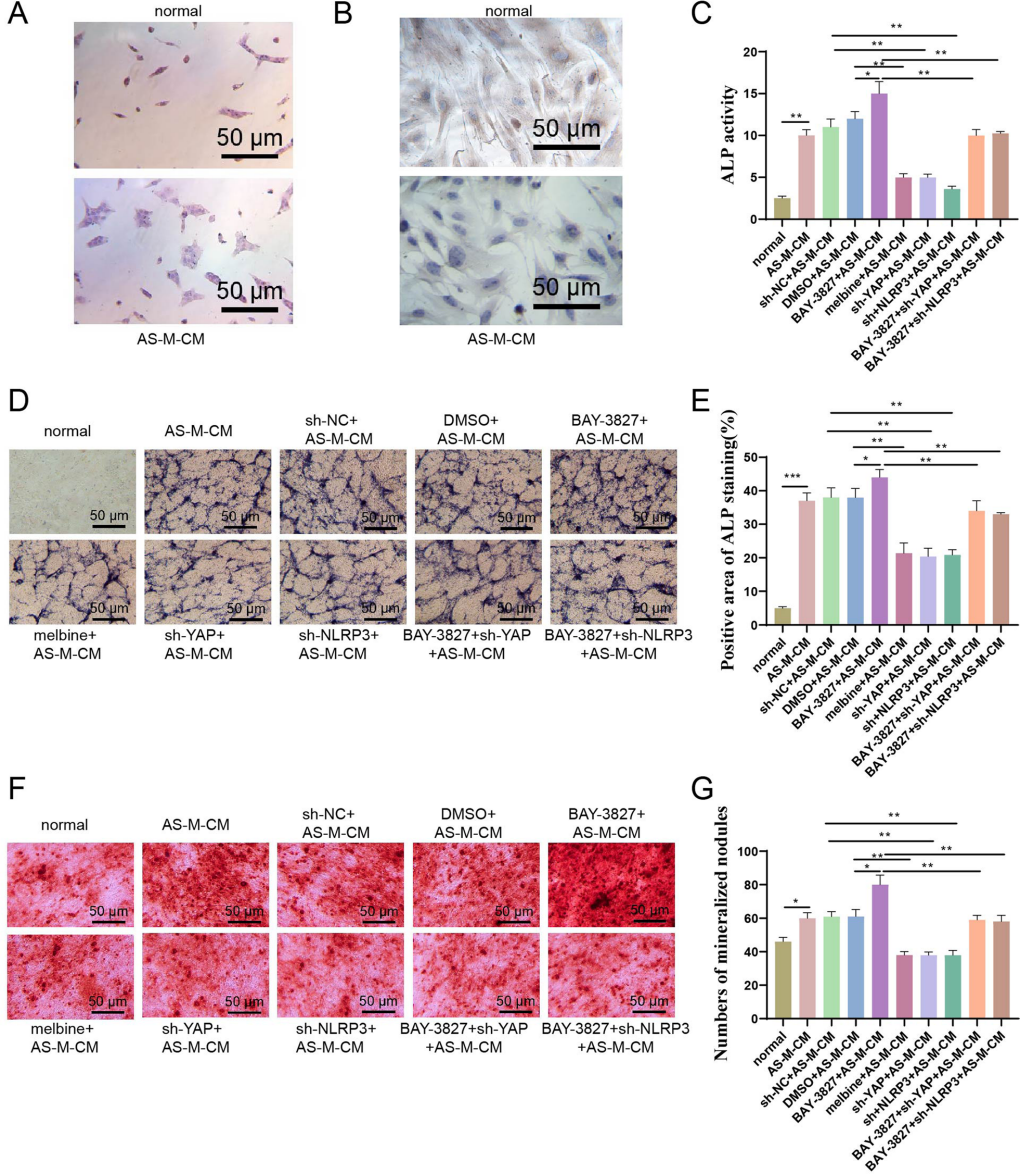
Effects of AMPK/YAP/NLRP3 signaling pathway on osteogenesis of fibroblasts. *Note* **A**, **B A** H&E staining and **B** Vimentin immunohistochemistry staining of normal fibroblasts and AS fibroblasts; Bar = 200 μm; **C** Detection of ALP activity in fibroblasts, normal for femoral neck fracture fibroblasts; **D** ALP staining, Bar = 200 μm; **E** Statistical chart of the percentage of ALP-positive cells in Figure **D**; **F** Safranin O staining, Bar = 200 μm; **G** Number of mineralized nodules formed in the statistical chart of Figure **F**. Values are presented as mean ± standard deviation. All cell experiments were repeated three times. *Indicates *P *< 0.05, **Indicates *P *< 0.01, and ***Indicates *P *< 0.001

The feature of AS is pathological bone formation [50]. Metformin is the most important activator of AMPK due to its anti-osteogenic effect on fibroblasts [38]. To verify if pro-inflammatory cytokines of monocytes have an impact on the calcification of fibroblasts. We first added the supernatant of mononuclear cells from AS patients (AS-M-CM) to human synovial fibroblasts (HSF) and co-cultured them for 48 h. The cells were then observed using H&E staining and immunohistochemistry staining with vimentin, a specific marker of fibroblasts. Cells detached from ligaments are elongated spindle-shaped or flattened stellate cells with oval-shaped nuclei and well-defined. The results showed that the cell morphology of femoral neck fracture patients (as normal controls) was normal (Fig. 4A), with positive Vimentin staining. In contrast, cells from the AS group exhibited a non-spindle shape and negative Vimentin staining (Fig. 4B), confirming that AS monocyte supernatant affects the osteogenesis of fibroblasts.

Next, we transfected AMPK inhibitor (BAY-3827), metformin, sh-YAP, and sh-NLRP3 into monocytes from AS patients and collected cell supernatant. Then, the supernatant was added to HSF cells and co-cultured for 48 h. Alkaline phosphatase (ALP) is one of the indicators of bone metabolism. Firstly, we quantitatively measured ALP to evaluate the osteogenic ability of HSF cells. The results showed that the ALP level in the AS-M-CM group significantly increased compared to the normal group. The ALP level significantly increased in the BAY-3827 + AS-M-CM group compared to the sh-DMSO + AS-M-CM group. The ALP level significantly decreased in the metformin + AS-M-CM group compared to the sh-DMSO + AS-M-CM group. However, knocking down YAP or NLRP3 led to a significant decrease in ALP activity in the AS-M-CM group compared to the sh-NC + AS-M-CM group. Adding sh-YAP or sh-NLRP3 to the BAY-3827 + AS-M-CM group further reduced ALP (Fig. 4C).

ALP (Fig. 4D, E) and Alizarin Red (Fig. 4F, G) staining were performed simultaneously, with Alizarin Red indicating the mineralized nodules produced by osteoblasts during bone formation. Consistent with the above quantitative ALP, normal fibroblasts have less positive ALP and eosinophilic staining. The results showed that after the suppression of AMPK, both ALP-positive staining and mineralized nodules increased. However, both of them decreased after knocking down YAP and NLRP3. Meanwhile, sh-YAP and sh-NLRP3 could be rescued by BAY-3827.

The above results indicate that inhibiting the secretion of inflammatory cytokines in mononuclear cells stimulated by AMPK will increase the ossification of fibroblasts while activating AMPK will alleviate the ossification of fibroblasts. Similarly, knocking down YAP or NLRP3 will reduce the ossification of fibroblasts by inhibiting pro-inflammatory factors.

### Knocking down NLRP3 could alleviate intervertebral disc degeneration in AS mice

To further clarify the expression of AMPK, YAP, and NLRP3 in AS through animal experiments in vivo, we induced AS mouse models using protein polysaccharides. After 18 weeks, we used CT to detect changes in the spine to determine whether the model was successfully induced. As shown in Additional file 3: Fig. S2A, the spine of the transgenic mice is significantly bent inward, and there are typical "bamboo-like changes" in the position of the intervertebral discs. In addition, the intervertebral space is narrowed, and the discs are significantly ossified. These changes are similar to the late-stage spinal fusion and stiffness seen in AS. After fixing the spine, decalcification, slicing, and staining were performed on the tissues. H&E and Safranin O-Fast green staining were used to examine the tissue sections. As shown in Additional file 3: Fig. S2B, the intervertebral disc of normal mice was obvious and intact, with no erosion or damage to the surrounding tissue. However, the intervertebral gap of the model mice was narrow, the intervertebral disc was damaged or even disappeared, and both ends showed fibrosis and ossification. AS mouse model induction was considered successful based on this judgment.

**Fig. 5:**
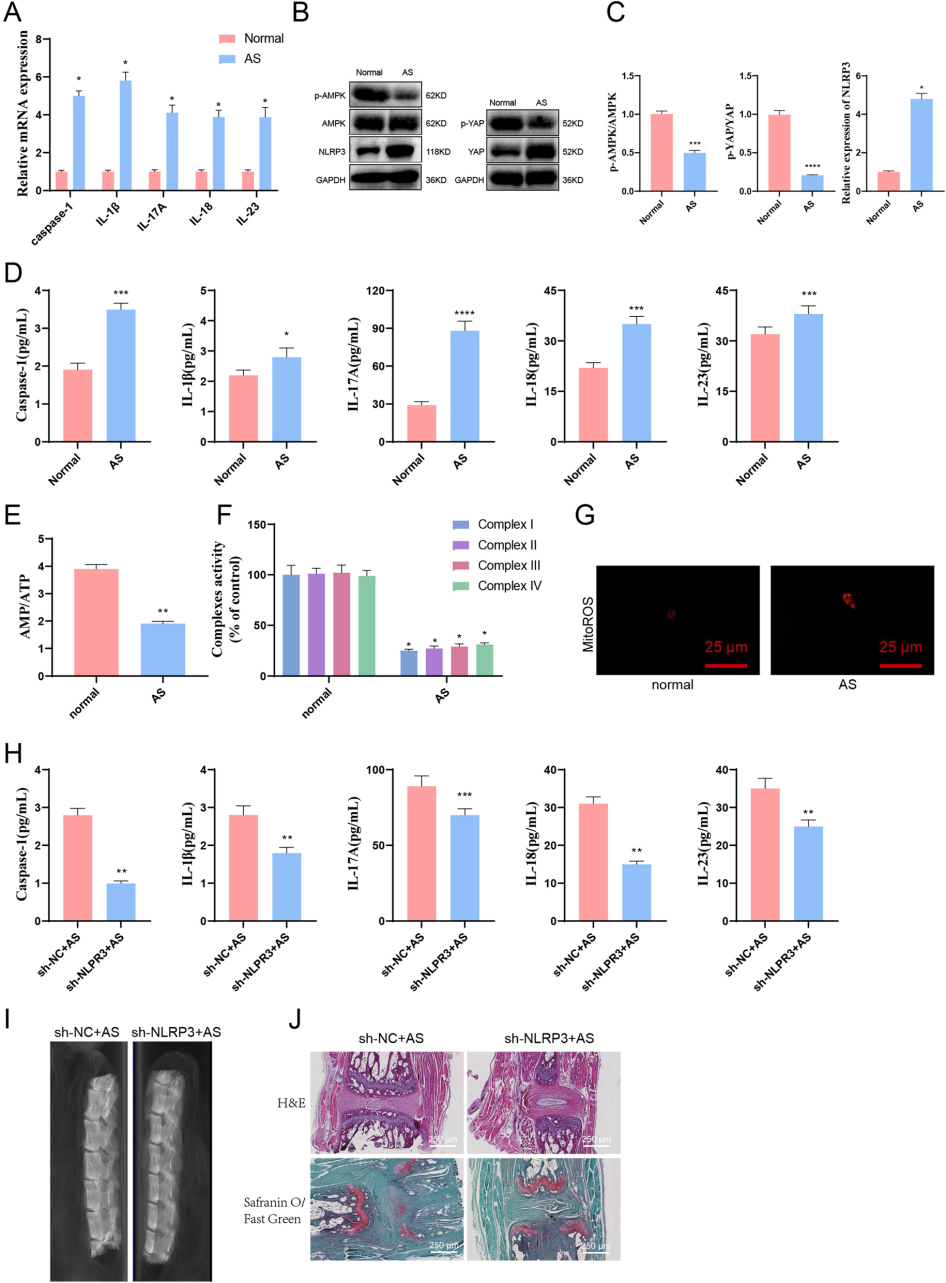
The effect of knocking down NLRP3 on AS mice. *Note* **A** Use RT-qPCR to detect the mRNA expression levels of caspase-1, IL-1β, IL-17A, IL-18, and IL-23 in the peripheral blood of AS mice and normal mice. Normal mice (normal) were used as controls, and GAPDH was used as the internal reference. **B** Use Western blot to detect the protein expression levels of AMPK, YAP, and NLRP3 in the peripheral blood of AS and normal mice. Normal mice (normal) were used as controls, and GAPDH was used as the internal reference. **C** The statistical analysis chart of B. Normal mice (normal) was used as control, and GAPDH was used as the internal reference. **D** ELISA was used to detect the levels of inflammatory factors caspase-1, IL-1β, IL-17A, IL-18, and IL-23 in the serum of AS and normal mice. Normal mice (normal) were used as controls. **E** Detect the ratio of AMP/ATP in peripheral blood mononuclear cells of mice. **F** The activity of mitochondrial complexes I-IV. **G** Observe mitochondrial ROS under laser confocal microscopy, Bar = 10 μm. **H** Inject sh-NLRP3 and sh-NC into the tail vein of AS mice, respectively, and detect the levels of inflammatory factors caspase-1, IL-1β, IL-17A, IL-18, and IL-23 by ELISA. The sh-NC was used as a control. **I** The cervical dislocation was used to euthanize mice, and the spine was dissected and cleaned for CT examination of bone changes in the spine. The arrow indicates the intervertebral disc space. **J** Stained the tissue near the intervertebral disc of mice with H&E and Safranin-O Fast Green stains (40 × , bar = 25 μm). Numerical values are presented as mean ± standard deviation; each group contains 6 mice, with *indicating *P *< 0.05, **indicating *P *< 0.01, and ***indicating *P *< 0.001 for comparisons between two groups

First, we euthanized AS mice and normal mice, took their joint tissues, ground them, extracted RNA and protein separately, and used RT-qPCR to detect the mRNA expression levels of caspase-1, IL-1β, IL-17A, IL-18, and IL-23 (Fig. 5A). Next, a western blot was used to detect the protein expression levels of AMPK, YAP, and NLRP3 (Fig. 5B). The results showed that the expression levels of caspase-1, IL-1β, IL-17A, IL-18, IL-23, YAP, and NLRP3 were significantly increased. Compared to the control group, the AMPK/AMPK and p-YAP/YAP of AS mice significantly decreased, indicating that the phosphorylation of AMPK and YAP were suppressed (Fig. 5C). Finally, ELISA was used to detect the inflammatory factors caspase-1, IL-1β, IL-17A, IL-18, and IL-23 in the peripheral blood serum of AS and normal mice. The results showed that compared to normal mice, the levels of inflammatory factors in the serum of AS mice were significantly increased (Fig. 5D).

We isolated mononuclear cells from the peripheral blood of mice and simultaneously measured the contents of AMP and ATP in mononuclear cells. The results showed that compared with normal mice, the AMP/ATP ratio of AS mice was significantly reduced (Fig. 5E). Meanwhile, AS inhibited the activity of the mitochondrial complex (Fig. 5F). Detection of mitochondrial reactive oxygen species (ROS) in AS mouse mononuclear cells revealed a significant increase in mitochondrial ROS compared to mononuclear cells from normal mice (Fig. 5G).

The above results indicate that in the AS mouse model, the AMPK signaling pathway is suppressed, the phosphorylation level of YAP is suppressed, and the expression level of NLRP3 is increased. To investigate the mechanism of NLRP3 in AS mice, we constructed two mouse-origin shRNA sequences of NLRP3 (sh-NLRP3-1 and sh-NLRP3-2). The knockdown efficiency was detected using RT-qPCR after injection into mice. The results showed that sh-NLRP3-2 had a higher knockdown efficiency. Therefore, sh-NLRP3-2 (after this, referred to as sh-NLRP3) was selected for subsequent experiments (Additional file 2: Fig. S1B).

After injecting protein-polysaccharide into mice for 7 days, we injected sh-NC and sh-NLRP3 into the tail vein of AS mice, respectively. Firstly, the expression level of NLRP3 in the spinal tissues of AS mice was detected. Knockdown of NLRP3 resulted in a significant decrease in NLRP3 expression in the spinal tissues of AS mice (Additional file 4: Fig. S3A, B). Next, ELISA was used to detect the levels of pro-inflammatory cytokines caspase-1, IL-1β, IL-17A, IL-18, and IL-23 in the peripheral blood serum of mice. The results showed that compared with the control AS mice, the levels of inflammatory factors in the serum of mice with NLRP3 knockdown were significantly reduced (Fig. 5H).

Using cervical dislocation to execute mice, stripping and washing the spine, and examining the bone changes of the spine with CT revealed that knocking down NLRP3 alleviated the intervertebral disc changes in mice. The mice's spines maintained thick intervertebral discs and normal curvature; no lumbar spinal stenosis occurred (Fig. 5I). H&E staining and safranin O-fast green staining were used to observe the changes in the mouse spine from a histological perspective. Safranin O-fast green staining mainly focused on the changes in cartilage. The results showed that knocking down NLRP3 could alleviate the destruction of intervertebral discs compared with the control AS mice, and the reduction of intervertebral disc cartilage was not significant (Fig. 5J). The above results indicate that knocking down NLRP3 could alleviate the symptoms of AS in mice while overexpressing NLRP3 could exacerbate the symptoms of AS in mice.

### Activation of AMPK or knockdown of YAP/NLRP3 could alleviate the erosion and destruction of AS spine in mice

The above experiments on AS patients and AS mice found that the AMPK signaling pathway was inhibited. To further evaluate the effect of AMPK on inflammation in AS mice, we injected AMPK inhibitors (BAY-3827) and AMPK activators (metformin) into the spine of AS mice. We assessed the inhibitory efficiency using RT-qPCR and Western blot techniques. The results showed that BAY-3827 significantly inhibited p-AMPK in AS mice, while metformin significantly activated p-AMPK in AS mice (Additional file 4: Fig. S3C).

**Fig. 6:**
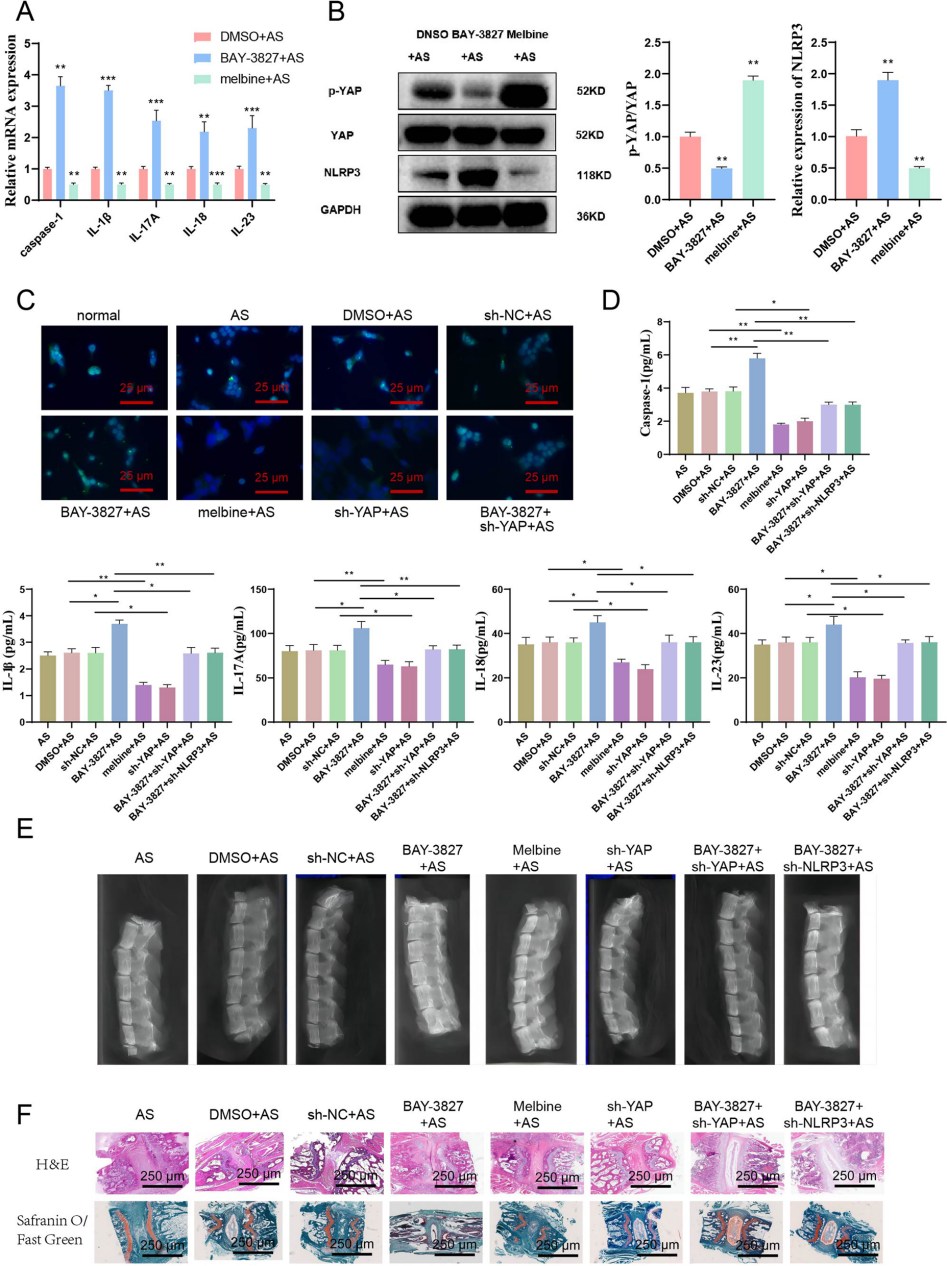
Mechanisms of the role of the AMPK/YAP/NLRP3 signaling pathway in the occurrence and development of AS validated by in vivo animal experiments. *Note* **A** BAY-3827 and metformin (timeline) were injected into the AS mouse joint separately, and RT-qPCR was used to detect the mRNA expression levels of caspase-1, IL-1β, IL-17A, IL-18, and IL-23 in mouse joint tissues. Sh-NC was used as a control, and GAPDH was used as an internal reference. **B** Western blot was used to detect the mRNA expression levels of YAP, p-YAP, and NLRP3 in mouse joint tissues. Sh-NC was used as a control. **C** Fluorescence microscopy was used to observe the formation of ASC specks. ASC: green, cell nucleus: blue, white arrows indicate ASC specks, bar = 10 μm. **D** BAY-3827, metformin (timeline), sh-YAP, and sh-NLRP3 were injected into the AS mouse joint separately, and ELISA was used to detect the inflammation factors caspase-1, IL-1β, IL-17A, IL-18, and IL-23. **E** The cervical dislocation was used to kill the mice, and the spine was removed, cleaned, and examined by CT for bone changes in the spine. The arrow points to the intervertebral disc space. **F** H&E staining and Safranin O-fast green staining were used to stain mice's tissues near the intervertebral disc (400 × , bar = 25 μm). The values are expressed as mean ± standard deviation. Each group includes 6 mice. *Indicates *P *< 0.05 for the comparison between two groups, **indicates *P *< 0.01, and ***indicates *P *< 0.001

BAY-3827, metformin, and DMSO (as a control) were injected into the spine of AS mice, and joint tissue from the mice was used to extract RNA and protein. RT-qPCR was used to detect the expression of inflammatory factors caspase-1, IL-1β, IL-17A, IL-18, and IL-23, while Western blot was used to detect the protein levels of p-YAP, YAP, and NLRP3. The results showed that inhibition of AMPK significantly increased the expression of caspase-1, IL-1β, IL-17A, IL-18, IL-23, YAP, and NLRP3 and significantly suppressed YAP phosphorylation. Conversely, activation of AMPK significantly reduced the expression of caspase-1, IL-1β, IL-17A, IL-18, IL-23, YAP, and NLRP3 and significantly increased YAP phosphorylation (Fig. 6A, B).

Design two sh-RNA sequences (sh-YAP-1 and sh-YAP-2) targeting YAP simultaneously. The knockdown efficiency was detected using RT-qPCR in fibroblast cells, and the results showed that sh-YAP-1 had higher efficiency. Therefore, sh-YAP-1 was chosen for subsequent experiments (referred to as sh-YAP hereafter) (Additional file 2: Fig. S1C, D). We injected sh-YAP into the spine of AS mice and detected the knockdown efficiency using RT-qPCR and Western blot. The results showed that sh-YAP significantly inhibited the expression of YAP in AS mice (Additional file 4: Fig. S3D, E).

First, we transfected BAY-3827, metformin, and sh-YAP into the separated AS mouse monocytes and detected the formation of ASC specks. The results showed that suppressing AMPK could increase the formation of ASC specks while activating AMPK could inhibit the formation. Furthermore, knocking down YAP also inhibits ASC speck formation, and sh-YAP could rescue BAY-3827-induced inhibition (Fig. 6C).

BAY-3827, metformin, sh-YAP, and sh-NLPR3 were injected into the spine of AS mice, and ELISA was used to detect the levels of caspase-1, IL-1β, IL-17A, IL-18, and IL-23 in the mouse serum. The results showed that BAY-3827 significantly increased the levels of inflammatory factors, while the levels of inflammatory factors further decreased after simultaneous injection of sh-YAP or sh-NLRP3 (Fig. 6D). The bone changes of AS mice spine were examined using CT. The results showed that compared with the sh-NC + AS group, inhibiting AMPK made the intervertebral space of mice narrower, and even fusion disappeared. The BAY-3827 + sh-YAP + AS and BAY-3827 + sh-NLRP3 + AS groups significantly relieved spine changes compared with the BAY-3827 + AS group (Fig. 6E). H&E staining and Safranin O-fast green staining results showed that compared to the control sh-NC + AS mice, the changes in intervertebral disc morphology were more obvious, and the intervertebral disc destruction was more significant after AMPK inhibition. There was a significant reduction in intervertebral disc cartilage. The destruction of the intervertebral disc was relieved in the BAY-3827 + sh-YAP + AS and BAY-3827 + sh-NLRP3 + AS groups compared with the BAY-3827 + AS group, and the reduction of intervertebral disc cartilage was not significant (Fig. 6F).

The above results show that inhibiting AMPK could exacerbate the AS symptoms in mice, significantly increase the expression of inflammatory factors and NLRP3, and reduce the phosphorylation level of YAP; activating AMPK could alleviate the erosion and damage of the AS spine in mice, significantly reduce the expression of inflammatory factors and NLRP3, increase the phosphorylation level of YAP, and knockdown of YAP or NLRP3 could reverse the effects caused by inhibiting AMPK.

## Discussion

In this study, we first discovered a significant correlation between NLRP3 and AS through bioinformatics analysis. NLRP3 activation could cause inflammation, and ATP could act as one of its activating factors [14]. Increasing ATPase activity could induce mitochondrial autophagy, replace defective mitochondria with new functional mitochondria, achieve the "purification effect" of mitochondria, and reduce ATP consumption. This process depends on AMPK signaling, thus activating AMPK signaling [21]. Changes in intracellular ATP levels could affect the transcriptional regulatory activity of YAP. Specifically, when ATP levels decrease, YAP is inhibited and its downstream signaling pathway is affected, thereby affecting cell proliferation and fate determination [19]. So we first hypothesized that the AMPK/YAP/NLRP3 pathway plays an important role in AS.

NLRP3 is an inflammasome protein complex in the body that plays a crucial role in immune defense. The NLRP3 complex comprises various proteins, including NLRP3 protein, ASC protein, and cysteine protease-1 (Caspase-1). When the body senses stimuli such as pathogens, intracellular signaling molecules, oxidative stress, etc., the NLRP3 complex is activated, activating Caspase-1 and the secretion of pro-inflammatory cytokines IL-1β and IL-18 [51]. The occurrence of AS is closely related to the NLRP3 inflammasome axis-associated factors NLRP3, caspase-1, ASC, IL-1β, IL-17A, and IL-23 [12]. Our research has found that the levels of pro-inflammatory factors, including caspase-1, IL-1β, IL-17A, IL-18, and IL-23, were significantly elevated in the peripheral blood of AS patients and AS mice. This further indicates that AS is closely related to the inflammatory response. Knocking down NLRP3 and activating AMPK significantly reduced the levels of pro-inflammatory factors, while inhibiting AMPK could significantly increase the levels of pro-inflammatory factors, suggesting that the activation of AMPK could alleviate the inflammatory response of AS.

ASC (apoptosis-associated speck-like protein containing a caspase recruitment domain) protein is a relatively simple structured protein containing a caspase recruitment domain (CARD) and a pyrin domain (PYD), and it is involved in the inflammatory response and immune reaction in various immune cells [52]. ASC speck is a small spot or cluster formed by polymeric ASC protein, typically found in immune cells such as monocytes and macrophages [53]. ASC protein aggregates to form a polymer during inflammation and complexes with NLRP3 protein to form NLRP3-ASC specks. This complex could induce the formation of inflammasomes, thereby triggering the inflammatory response. Therefore, ASC speck is an important component of inflammation in immune cells, and its formation and degradation process is crucial for regulating immune and inflammatory responses [54]. Therefore, in this study, fluorescent microscopy was used to observe the formation of ASC specks in single cells. The results showed that inhibiting AMPK could increase the formation of ASC specks, while activating AMPK could suppress the formation of ASC specks, further indicating that AMPK inhibits the activation of NLRP3 inflammasome.

As the main organelle of eukaryotic cells, mitochondria account for about one-fifth of the cell volume. They are widely involved in regulating cellular energy metabolism, proliferation, differentiation, aging, and death. Mitochondrial dysfunction is associated with the pathological progression of osteoarthritis through various pathways, including oxidative stress [55]. Mechanistically, excessive mitochondrial fission could lead to mitochondrial dysfunction, such as loss of mitochondrial DNA integrity, decreased ATP generation, and mitochondrial ROS burst [56]. Furthermore, ATP is inseparable from AMPK, YAP, and NLRP3. We have demonstrated through experiments that the ATP-mitochondrial complex is significantly increased in patients with AS and AS mice.

Existing studies have shown that AMPK could inhibit the activation of NLRP3 inflammasome, reduce the production of IL-1β and IL-18, and thus suppress inflammatory response [23]. NLRP3 could perceive danger signals inside and outside cells and activate signaling pathways such as NF-κB. AMPK could also be anti-inflammatory by inhibiting the activation of NF-κB and reducing ROS production [57]. When the body is infected or injured, the inflammatory response could induce the activation and translocation of YAP and then regulate the occurrence and progression of the inflammatory response. By inhibiting the expression or function of YAP, the level of inflammatory mediators could be reduced, and the tissue damage caused by the inflammatory response could be alleviated [58]. YAP's expression level in AS has significantly increased, especially during inflammation and bone tissue reconstruction processes. The YAP signaling pathway could promote the expression and activation of NLRP3, thus triggering inflammatory and immune responses, by activating the nuclear factor κB (NF-κB) pathway and enhancing the production of reactive oxygen species (ROS) [24, 59]. AMPK could phosphorylate YAP, causing it to bind to 14–3–3 protein and leading to its translocation from the nucleus to the cytoplasm, thereby inhibiting YAP's transcriptional activity [19, 22].

Moreover, research has shown that resveratrol reduces mouse AS by inhibiting the TLR4/NF-κB/NLRP3 pathway and regulating the intestinal microbiota [40].

Similarly, resveratrol inhibits the expression of YAP by activating AMPK [49]. Our experiment found that the expression levels of YAP and NLRP3 were significantly increased in AS patients and AS mice, and the phosphorylation of YAP was inhibited. Knocking down AMPK in AS mice significantly increases the expression of YAP and NLRP3 and significantly reduces phosphorylated YAP. The above results indicate that AMPK could promote the phosphorylation of YAP.

YAP could regulate the NLRP3 pathway by participating in its ubiquitination to influence the inflammatory response. Specifically, the YAP signaling pathway could inhibit the K27 ubiquitination modification of NLRP3, thereby promoting the activation of NLRP3 and the degree of the inflammatory response [24]. We verified through ubiquitination experiments that YAP could inhibit K27 ubiquitination of NLRP3, and overexpression of AMPK could block YAP's inhibition of NLRP3 ubiquitination, thereby reducing the expression of NLRP3.

AS is an autoimmune disease characterized by pathological bone formation, inflammatory back stiffness, and pain [50]. As the main cells in connective tissue, fibroblasts contribute to heterotopic ossification during the process [60]. Research has shown that metformin could inhibit the ossification of fibroblasts through its anti-inflammatory effects on the Pi3k/Akt and AMPK pathways [38]. We co-cultured monocyte-derived macrophage supernatant of AS patients with fibroblasts and found that AMPK could affect fibroblast ossification by inhibiting the inflammatory response of monocytes.

AS is an autoimmune disease characterized primarily by chronic spine inflammation and joint stiffness. Patients with AS often experience symptoms of intervertebral discitis, such as disc deformation, collapse, and displacement. The vertebral bone typically becomes fragile, while the facet joints may become inflamed and destroyed, resulting in joint stiffness and spinal deformity [61]. In this study, we induced AS-like symptoms in BALB/c mice using protein-polysaccharide and confirmed the consistency of symptoms with AS through CT and tissue staining. The experiment demonstrated that knocking down NLRP3 could alleviate symptoms in AS mice while knocking down AMPK could worsen joint lesions. Simultaneously knocking down both AMPK and NLRP3 did not result in significant changes in symptoms compared to the control AS mice, indicating that knocking down NLRP3 could compensate for the effects of knocking down AMPK.

**Fig. 7:**
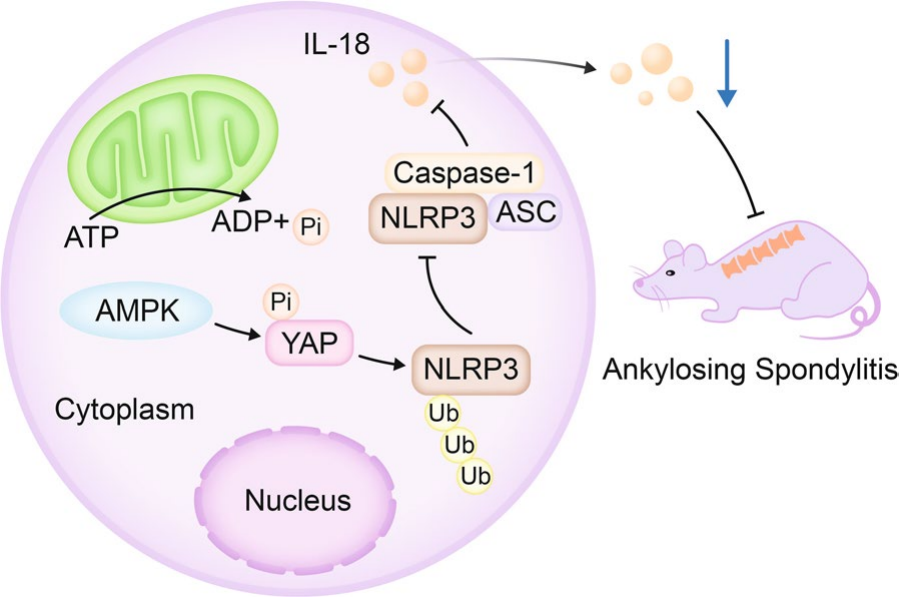
Schematic diagram of the molecular mechanisms by which the AMPK/YAP/NLRP3 signaling pathway affects the occurrence and development of ankylosing spondylitis

In summary, we could preliminarily draw the following conclusion that AMPK may suppress YAP expression to increase NLRP3 ubiquitination and degradation, thereby inhibiting the activation of NLRP3 inflammasome and ultimately alleviating AS (Fig. 7). However, although this study has achieved certain results in exploring the role of the AMPK/YAP/NLRP3 signaling pathway in the pathogenesis of AS, there are still limitations. Firstly, this study only collected 30 peripheral blood samples from AS patients and 30 normal controls, which is relatively small and may affect the stability and representativeness of the research results. Secondly, although AS mouse models are effective tools for studying the pathogenesis of AS and evaluating treatment strategies, there are still differences between mouse models and humans regarding physiology and pathology. Therefore, research results based on mouse models must be validated in clinical trials to ensure their applicability in humans.

## Conclusion

Furthermore, this study mainly focuses on the role of the AMPK/YAP/NLRP3 signaling pathway in the pathogenesis of AS. However, the pathogenesis of AS is complex and may involve other signaling pathways that have not been explored. Therefore, it is necessary to further investigate the role of other signaling pathways in the pathogenesis of AS to fully understand the pathological mechanism of the disease. In summary, future research needs to expand sample sizes, consider population specificity, validate the applicability of experimental models, conduct long-term follow-ups, and explore other signaling pathways to comprehensively understand the pathogenesis of ankylosing spondylitis and optimize treatment strategies.

### Supplementary Information

**Additional file 1: Table S1.** RT-qPCR primer sequence (Human). **Table S2.** RT-qPCR primer sequence (Mouse).

**Additional file 2: Fig. S1.** Comparison of knockdown efficiency among different shRNA sequences. *Note* Transfect sh-NC, sh-YAP, and sh-NLRP3 into monocytes and use RT-qPCR to detect knockdown efficiency of human YAP (**A**), human NLRP3 (**B**), mouse NLRP3 (**C**), and mouse YAP (**D**). Values are presented as mean ± standard deviation. All experiments were repeated three times. *Denotes *P* < 0.05, **denotes *P* < 0.01.

**Additional file 3: Fig. S2.** Identification of AS mouse model. *Note* **A** Proteoglycan was used to induce AS mouse model, and changes in the spine were detected by CT 18 weeks later, with arrows pointing to the intervertebral disc spaces. **B** H&E staining and Safranin O-fast green staining were used to stain the tissues near the intervertebral discs of normal mice (normal) and AS mice (40×, bar = 25 μm); each group contained 6 mice.

**Additional file 4: Fig. S3.** NLRP3, AMPK, and YAP expression levels in AS mouse spine tissues were detected. **B** In AS mice, sh-NLRP3 and sh-NC were transfected, and the expression level of NLRP3 in the mouse spine was detected by RT-qPCR (**A**) and Western blot (**B**); **C** BAY-3827, metformin (timeline), and DMSO were injected into AS mice, and the expression levels of p-AMPK and AMPK in the mouse spine were detected by Western blot (**C**); **D**, **E**: sh-YAP and sh-NC were transfected in AS mice, and the expression level of YAP in the mouse spine was detected by RT-qPCR (**D**) and Western blot (**E**). Values are presented as mean ± standard deviation, all experiments were repeated three times, and **indicates *P *< 0.01 for comparison between the two groups. The values are expressed as mean ± standard deviation; there are 6 mice per group; **indicates *P* < 0.01 for comparison between the two groups

## Data Availability

The datasets used and/or analyzed during the current study are available from the corresponding author on reasonable request.

## Abbreviations

AS: Ankylosing spondylitis
AMPK: AMP-activated protein kinase
GO: Gene ontology
KEGG: Kyoto Encyclopedia of Genes and Genomes
ELISA: Enzyme-linked immunosorbent assay
