# Supplementary material for: Molecular mechanisms of AMPK/YAP/NLRP3 signaling pathway affecting the occurrence and development of ankylosing spondylitis
Source: J Orthop Surg Res. 2023 Nov 4;18:831. doi: 10.1186/s13018-023-04200-x (PMC10625209; doi:10.1186/s13018-023-04200-x)
Supplement: Supplementary file 1 — Additional file 1: Table S1. RT-qPCR primer sequence (Human). Table S2. RT-qPCR primer sequence (Mouse). [file 13018_2023_4200_MOESM1_ESM.docx]

**Table S1. RT-qPCR primer sequence (Human)**

| Gene | Sequence (human) |
| --- | --- |
| AMPK | F: 5'-GACAGCCGAGAAGCAGAAAC-3' |
|  | R: 5'-AGGATGCCTGAAAAGCTTGA-3' |
| Caspase-1 | F: 5'-GCTTTCTGCTCTTCCACACC-3' |
|  | R: 5'-CATCTGGCTGCTCAAATGAA-3' |
| IL1-β | F: 5'-GGGCCTCAAGGAAAAGAATC-3' |
|  | R: 5'-TTCTGCTTGAGAGGTGCTGA-3' |
| IL-17A | F: 5'-ACCAATCCCAAAAGGTCCTC-3' |
|  | R: 5'-GGGGACAGAGTTCATGTGGT-3' |
| IL-23 | F: 5'-GTTCCCCATATCCAGTGTGG-3' |
|  | R: 5'-GGCTATCAGGGAGCAGAGAA-3' |
| YAP | F: 5'-GCAGTTGGGAGCTGTTTCTC-3' |
|  | R: 5'-GCCATGTTGTTGTCTGATCG-3' |
| NLRP3 | F: 5'-CTTCTCTGATGAGGCCCAAG-3' |
|  | R: 5'-GCAGCAAACTGGAAAGGAAG-3' |
| GAPDH | F: 5'-GGCTGTTGTCATACTTCTCATGG-3' |
|  | R: 5'-GGAGCGAGATCCCTCCAAAAT-3' |

Note: F, forward; R, reverse.

**Table S2. RT-qPCR primer sequence (Mouse)**

| Gene | Sequence (mouse) |
| --- | --- |
| AMPK | F: 5'-AGAGGGCCGCAATAAAAGAT-3' |
|  | R: 5'-TGTTGTACAGGCAGCTGAGG-3' |
| Caspase-1 | F: 5'-GATGGCACATTTCCAGGACT-3' |
|  | R: 5'-GATCCTCCAGCAGCAACTTC-3' |
| IL1-β | F: 5'-GGGCCTCAAAGGAAAGAATC-3' |
|  | R: 5'-TACCAGTTGGGGAACTCTGC-3' |
| IL-17A | F: 5'-TCTCTGATGCTGTTGCTGCT-3' |
|  | R: 5'-CGTGGAACGGTTGAGGTAGT-3' |
| IL-23 | F: 5'-AATAATGTGCCCCGTATCCA-3' |
|  | R: 5'-CATGGGGCTATCAGGGAGTA-3' |
| YAP | F: 5'-AGGAGAGACTGCGGTTGAAA-3' |
|  | R: 5'-CCTGAGACATCCCAGGAGAA-3' |
| NLRP3  GAPDH | F: 5'- AGGCTGCTATCTGGAGGAACT-3' |
|  | R: 5'- CCTTTCTCGGGCGGGTAATC-3'  F: 5'-TGATGGGTGTGAACCACGAG-3'  R: 5'-AGTGATGGCATGGACTGTGG-3' |

Note: F, forward; R, reverse.
