# Supplementary figures and images for: Molecular mechanisms of AMPK/YAP/NLRP3 signaling pathway affecting the occurrence and development of ankylosing spondylitis
Source: J Orthop Surg Res. 2023 Nov 4;18:831. doi: 10.1186/s13018-023-04200-x (PMC10625209; doi:10.1186/s13018-023-04200-x)

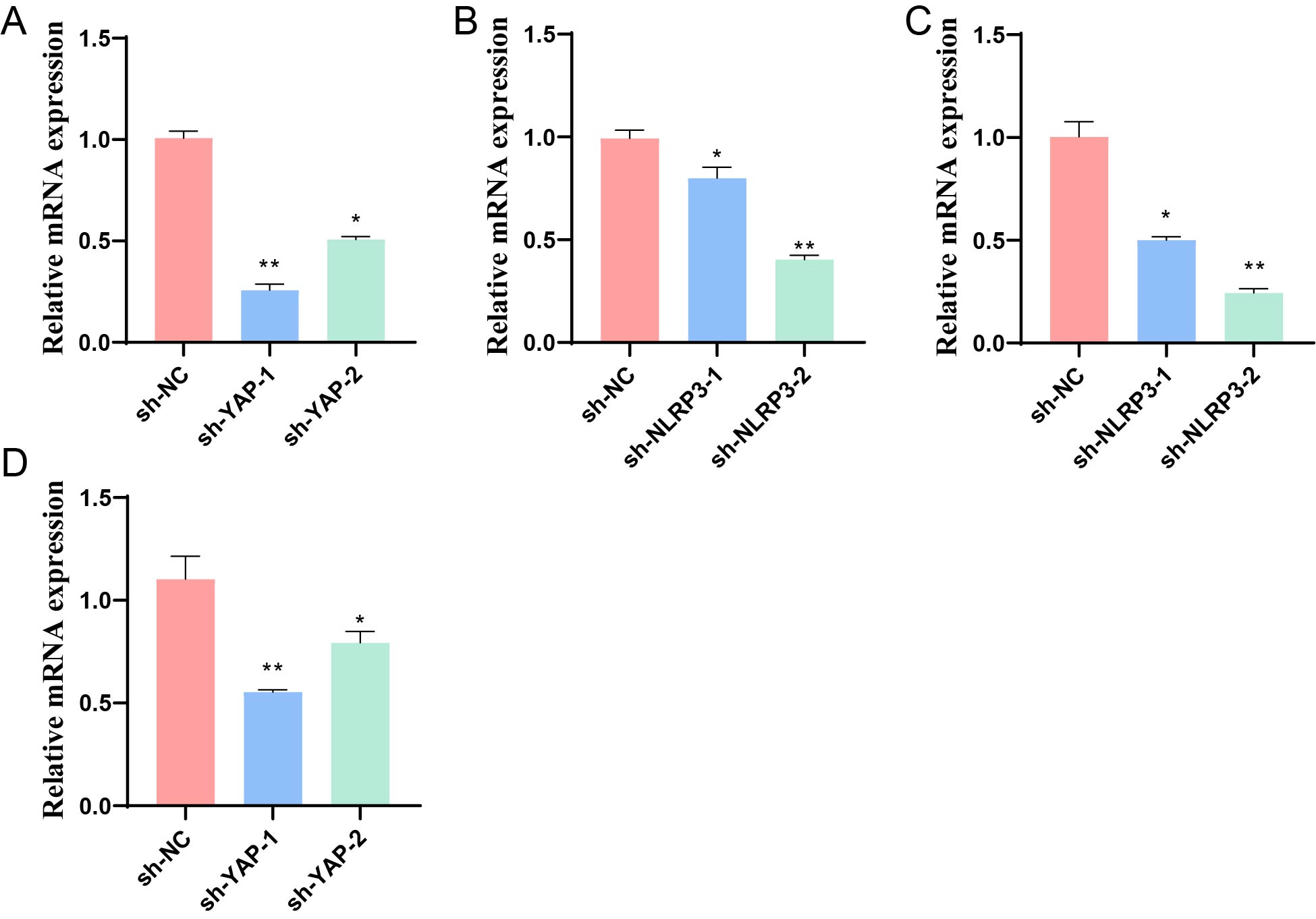

Supplement: Supplementary file 2 — Additional file 2: Fig. S1. Comparison of knockdown efficiency among different shRNA sequences. Note Transfect sh-NC, sh-YAP, and sh-NLRP3 into monocytes and use RT-qPCR to detect knockdown efficiency of human YAP (A), human NLRP3 (B), mouse NLRP3 (C), and mouse YAP (D). Values are presented as mean ± standard deviation. All experiments were repeated three times. *Denotes P < 0.05, **denotes P < 0.01. [file 13018_2023_4200_MOESM2_ESM.jpg]

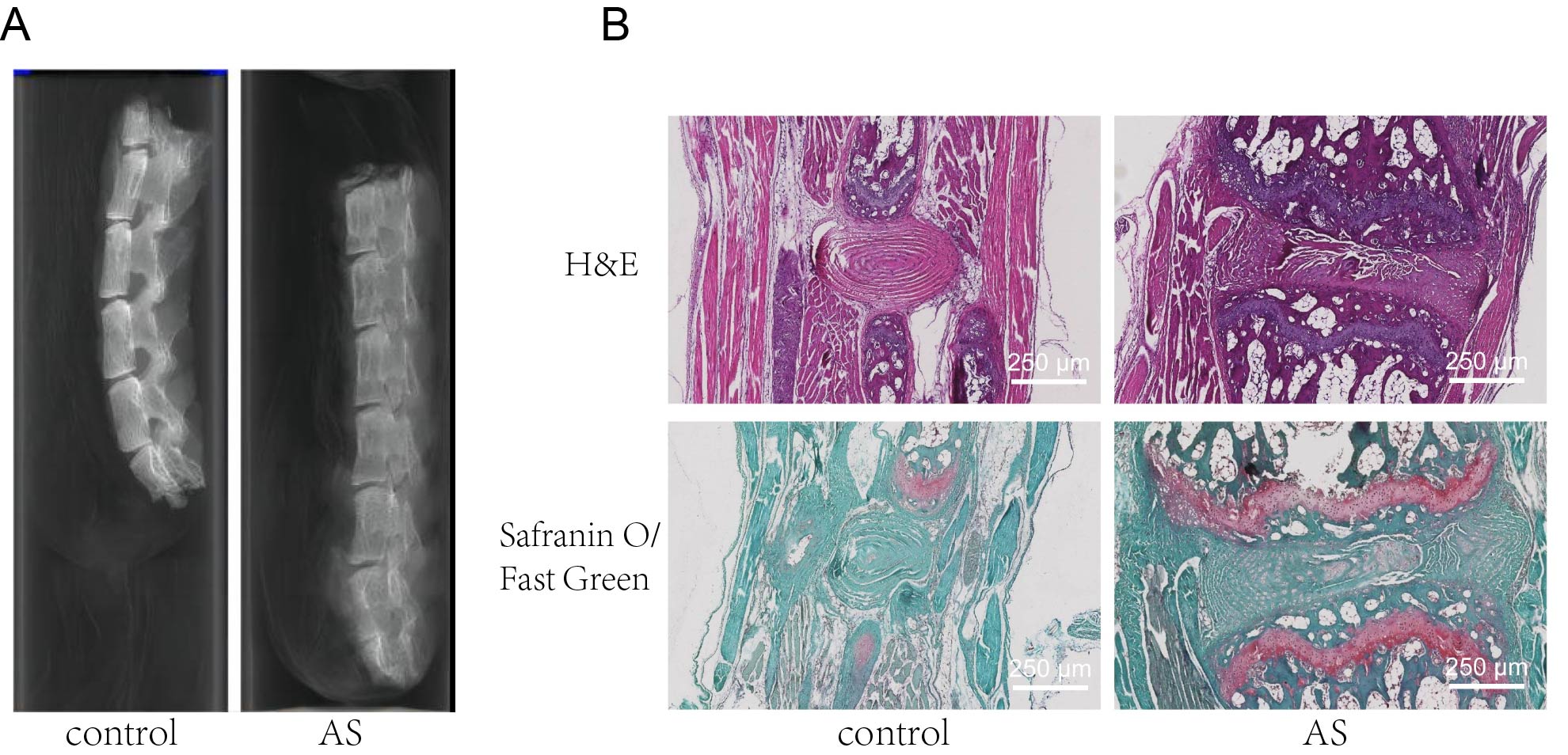

Supplement: Supplementary file 3 — Additional file 3: Fig. S2. Identification of AS mouse model. Note A Proteoglycan was used to induce AS mouse model, and changes in the spine were detected by CT 18 weeks later, with arrows pointing to the intervertebral disc spaces. B H&E staining and Safranin O-fast green staining were used to stain the tissues near the intervertebral discs of normal mice (normal) and AS mice (40×, bar = 25 μm); each group contained 6 mice. [file 13018_2023_4200_MOESM3_ESM.jpg]

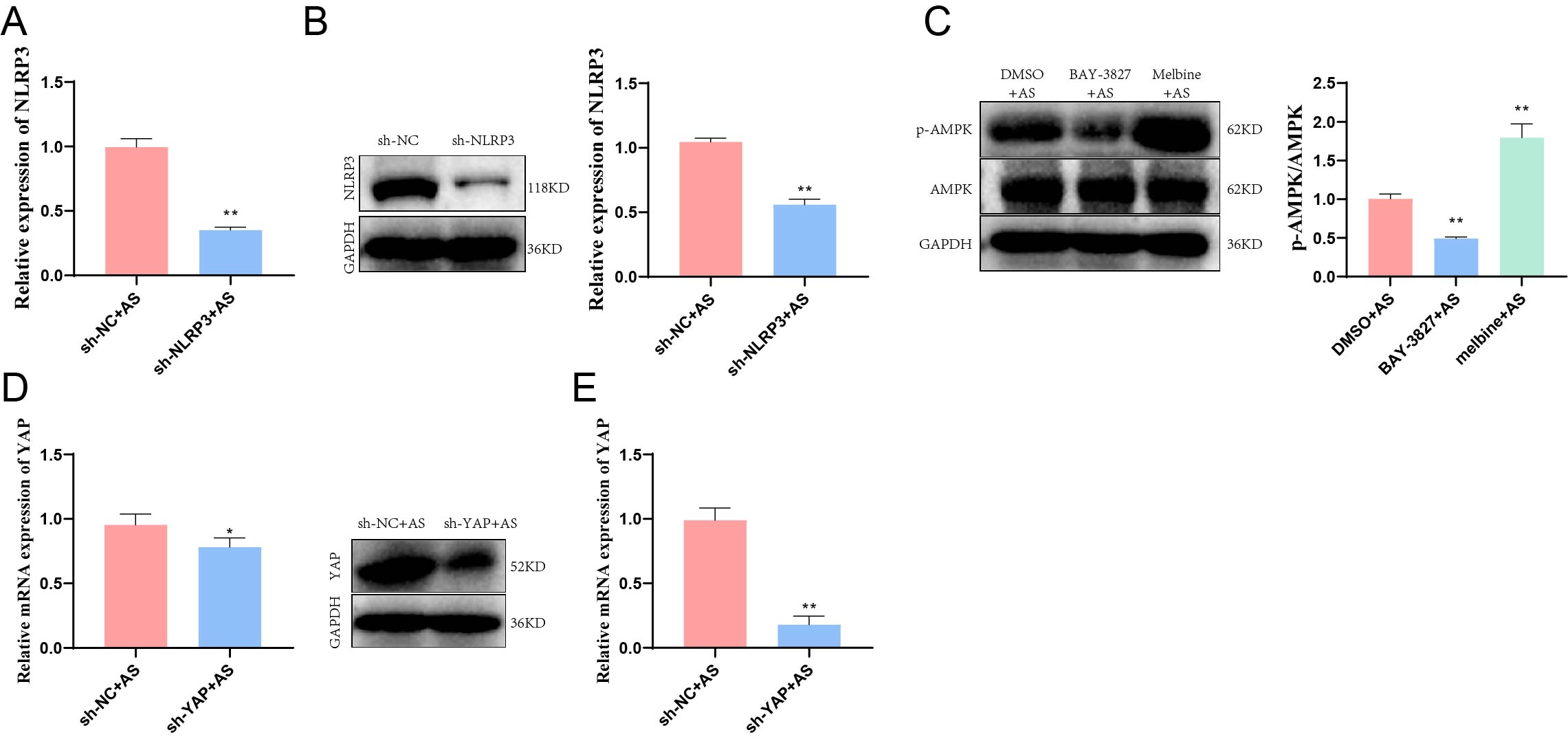

Supplement: Supplementary file 4 — Additional file 4: Fig. S3. NLRP3, AMPK, and YAP expression levels in AS mouse spine tissues were detected. B In AS mice, sh-NLRP3 and sh-NC were transfected, and the expression level of NLRP3 in the mouse spine was detected by RT-qPCR (A) and Western blot (B); C BAY-3827, metformin (timeline), and DMSO were injected into AS mice, and the expression levels of p-AMPK and AMPK in the mouse spine were detected by Western blot (C); D, E: sh-YAP and sh-NC were transfected in AS mice, and the expression level of YAP in the mouse spine was detected by RT-qPCR (D) and Western blot (E). Values are presented as mean ± standard deviation, all experiments were repeated three times, and **indicates P < 0.01 for comparison between the two groups. The values are expressed as mean ± standard deviation; there are 6 mice per group; **indicates P < 0.01 for comparison between the two groups [file 13018_2023_4200_MOESM4_ESM.jpg]
